# Retinitis Pigmentosa: Current Clinical Management and Emerging Therapies

**DOI:** 10.3390/ijms24087481

**Published:** 2023-04-19

**Authors:** Xuan-Thanh-An Nguyen, Lude Moekotte, Astrid S. Plomp, Arthur A. Bergen, Maria M. van Genderen, Camiel J. F. Boon

**Affiliations:** 1 Department of Ophthalmology, Leiden University Medical Center, 2333 ZA Leiden, The Netherlands; 2 Department of Ophthalmology, University Medical Center Utrecht, 3584 CX Utrecht, The Netherlands; 3 Department of Clinical Genetics, Amsterdam University Medical Centers, Meibergdreef 9, 1105 AZ Amsterdam, The Netherlands; 4 Bartiméus, Diagnostic Center for Complex Visual Disorders, 3703 AJ Zeist, The Netherlands; 5 Department of Ophthalmology, Amsterdam University Medical Centers, Meibergdreef 9, 1105 AZ Amsterdam, The Netherlands

**Keywords:** retinitis pigmentosa, clinical management, genetics, genetic counseling, gene therapy, low vision, low-vision rehabilitation

## Abstract

Retinitis pigmentosa (RP) comprises a group of inherited retinal dystrophies characterized by the degeneration of rod photoreceptors, followed by the degeneration of cone photoreceptors. As a result of photoreceptor degeneration, affected individuals experience gradual loss of visual function, with primary symptoms of progressive nyctalopia, constricted visual fields and, ultimately, central vision loss. The onset, severity and clinical course of RP shows great variability and unpredictability, with most patients already experiencing some degree of visual disability in childhood. While RP is currently untreatable for the majority of patients, significant efforts have been made in the development of genetic therapies, which offer new hope for treatment for patients affected by inherited retinal dystrophies. In this exciting era of emerging gene therapies, it remains imperative to continue supporting patients with RP using all available options to manage their condition. Patients with RP experience a wide variety of physical, mental and social-emotional difficulties during their lifetime, of which some require timely intervention. This review aims to familiarize readers with clinical management options that are currently available for patients with RP.

## 1. Introduction

Retinitis pigmentosa (RP) is a collective term used to describe a heterogeneous group of inherited retinal dystrophies (IRDs) that are characterized by primary loss of rod photoreceptors, followed by secondary loss of cone photoreceptors [1]. This degenerative process leads to a gradual loss in visual function in affected individuals and may ultimately lead to loss of visual functions in more advanced stages [1,2]. RP has a variable prevalence of 1 in 750–9000 individuals, depending on the geographic location of the reported study [1,2,3,4,5,6,7,8]. Higher incidences of RP are typically found in regions with high rates of consanguinity and in (semi-)isolated populations [7,9]. In the Western population, the global prevalence of RP has been estimated to be around 1 in 3000–5000 individuals [1,2].

The term ‘retinitis pigmentosa’ was first introduced by Dutch physician F.C. Donders in 1857, after a few previous reports of possible RP, including potentially the first fundus drawing of a patient with RP (or choroideremia) by Dutch physician Van Trigt, a PhD student of Donders, soon after the introduction of the ophthalmoscope [10,11,12,13]. While the term ‘retinitis pigmentosa’ is considered a misnomer, it is still widely used in clinical and academic settings [1]. The term ‘rod–cone dystrophy’ is often used interchangeably with RP, as it denotes the order of photoreceptor degeneration occurring in this retinal disease [14,15,16,17].

While RP was previously considered to be untreatable, promising medical advances, particularly the development of genetic therapies, have paved the way for potential therapies that may slow down or halt photoreceptor degeneration, or even restore some degree of visual function [18]. Our improved understanding of the cellular mechanisms and genetic background underlying IRDs, along with the immune-privileged characteristics of the eye, has heralded gene therapy as one of the most promising therapies for RP [19]. Proof-of-concept studies in murine and canine models have shown the potential of gene therapy for *RPE65*-associated retinopathy, which has led to the initiation of human clinical gene-therapy trials [20,21,22,23,24]. The positive results in both safety profiles and clinical endpoints in these trials have resulted in the approval of voretigene neparvovec as the first FDA-approved gene therapy for patients with *RPE65*-associated retinopathy, which is now commercially available as Luxturna^®^ [20,25,26,27]. This major milestone has sparked a surge in interest for other IRDs and their candidate genes, and several gene-therapy clinical trials have already commenced and terminated [19,27,28,29,30,31].

Despite rapid developments in genomic medicine, some important considerations remain for the implementation of these therapeutic strategies. For instance, while gene therapy holds promise for patients with IRDs, not every patient will be eligible for this treatment. Generally, genetic therapies require identification of the causative gene and photoreceptor viability in order to be effectively applied [28]. Many IRD patients do not meet both of these criteria for gene therapy. As such, patients require therapy tailored to their genetic condition and disease stage, or a therapy that can be universally applied regardless of the underlying genetic cause [18,25,28]. Furthermore, when designing a clinical trial, chosen outcome measures need to be relevant and meaningful for the intended retinal disease and patient [32]. As RP is mostly a progressive, degenerative disease, timely intervention would provide the most benefit [33].

Additionally, RP is associated with an increased risk of other ocular complications, such as cataract and cystoid macular edema (CME), which may cause additional visual disturbances [34,35]. The combination of RP with other potentially vision-impairing complications often causes significant visual impairment at an early age, which also impacts a patient’s physical and mental health [36]. Currently, several management options for RP exist, ranging from genetic and psychological counseling to the treatment of RP-associated complications. Although these management options are considered supportive, they certainly provide some relief of the physical, mental and social-emotional burden that patients may experience [37].

In this review, we aim to update and familiarize readers with the current tools for the clinical management of RP, as new management modalities have become available over the years. This information can be used by clinicians to provide patients with updated insights into current management options, to weigh their benefits and drawbacks, and in turn, advise patients in the management of their disease.

## 2. Pathophysiology of RP

**Figure 1:**
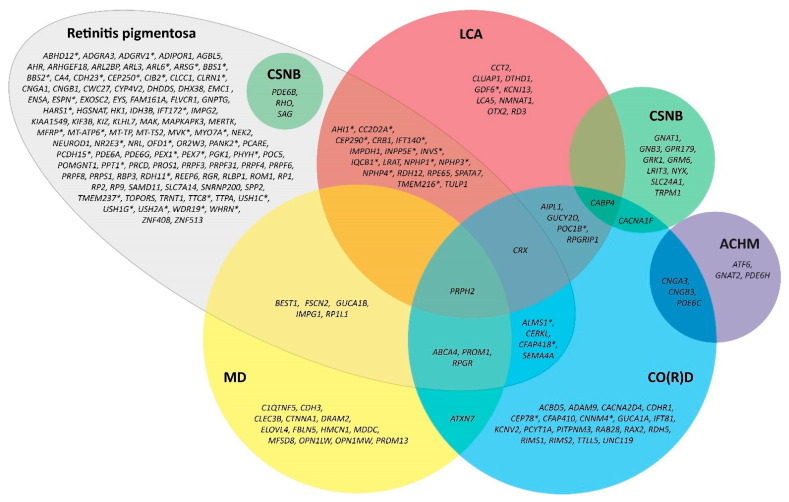
Venn diagram of currently identified genes associated with retinitis pigmentosa (RP) and their genetic overlap with other inherited retinal dystrophies. For example, variants in the *RHO* gene can manifest in either RP or congenital stationary night-blindness phenotypes. All genes included are registered in the Online Mendelian Inheritance in Man (OMIM) database and follow the up-to-date symbols of the HUGO Gene Nomenclature Committee (HGNC). Genes that are associated with syndromic forms of RP are marked with an asterisk (*). ACHM = achromatopsia; CO(R)D = cone(-rod) dystrophy; CSNB = congenital stationary night blindness; LCA = Leber Congenital Amaurosis; MD = macular dystrophy.

RP is mostly a monogenic disease, in which most disease-associated genetic variants are expressed in photoreceptor or retinal pigment epithelium (RPE) cells, although digenic inheritance has also been described [1,38]. As each gene has its own function, genetic variants lead to different biochemical changes within the retina. Eventually, these changes result in the degeneration of photoreceptors and RPE cells. To date, more than 90 genes have been linked to RP, and it is likely that this number will increase over the years due to ongoing improvements in diagnostic testing techniques (RetNet, https://sph.uth.edu/RetNet/; accessed on 1 November 2022) [1,39,40]. RP is a highly heterogeneous disease, both clinically and genetically, and shows considerable overlap with other IRDs. Identical disease-associated genetic variants may manifest in different clinical entities, whereas different variants in different genes may also result in similar phenotypes [40]. An overview of the different causative genes in RP and their overlap with other IRDs is shown in Figure 1.

## 3. Classification of RP

### 3.1. Mode of Inheritance

RP comprises a spectrum of retinal phenotypes, some of which may exhibit unique clinical characteristics. Several classification systems have been proposed. The most common method to classify patients with RP is by their Mendelian mode of inheritance. RP can be inherited as autosomal dominant (adRP; 15–25%), autosomal recessive (arRP; 5–20%) or X-linked recessive (XLRP; 5–15%) [1,41]. Other inheritance patterns for RP, albeit very rare, also exist, namely in X-linked dominant, mitochondrial, and digenic forms [14]. Patients with no positive family history or definitive molecular diagnosis are termed isolated or simplex cases. These simplex cases are assumed to be primarily autosomal recessive, although other inheritance forms are also conceivable [42].

### 3.2. Non-Syndromic and Syndromic Forms of RP

RP can also manifest with extra-ocular symptoms, which occurs in 20–30% of all cases [1,14]. The most common extra-ocular symptom in combination with RP is hearing loss, in the context of Usher syndrome [43]. Patients are classified into ‘syndromic RP’ or ‘non-syndromic RP’ categories based on the distinction of whether extra-ocular features are present or absent, respectively. Additionally, most patients with syndromic RP can be further classified into either ‘inborn errors of metabolism (IEM)’ or ‘ciliopathies’ [44].

IEM includes a large group of genetic disorders in which the function of a crucial enzyme in one of the metabolic pathways is lost (e.g., carbohydrate, protein, or glycogen storage pathways) [44]. IEM has a predilection for the brain, and in turn can also affect the retina as it is part of the central nervous system [44]. Examples include adult Refsum disease (RP, neurodegeneration, ataxia, hearing loss, anosmia, and cardiac/skeletal/skin involvement), Bassen–Kornzweig syndrome (RP, fat malabsorption, acanthocytosis, low blood cholesterol, neurodegeneration) and PHARC syndrome (polyneuropathy, hearing loss, ataxia, RP and cataract) [45,46,47,48,49].

Ciliopathies are a group of disorders that affect the assembly or function of primary cilia. Cilia are microtubular extensions of the plasma membrane and are a component of nearly every cell type. As a consequence, genetic defects in the cilia are typically pleiotropic, affecting more than one system [44]. In the retina, the proximal end of the photoreceptors’ outer segments is connected to their inner segments via the connecting cilium. Other organs that are often affected in ciliopathies are the inner ear, kidney, liver, and central nervous system [44]. Known ciliopathies that can manifest with retinal degeneration include Usher syndrome, Joubert syndrome (retinal degeneration, intellectual disability, polydactyly, ataxia), Senior–Loken Syndrome (retinal degeneration and nephronophthisis) and Bardet–Biedl syndrome (RP, intellectual disability, polydactyly, obesity, and hypogonadism), among others [44,46,47,50,51,52].

## 4. Clinical Symptoms

**Figure 2:**
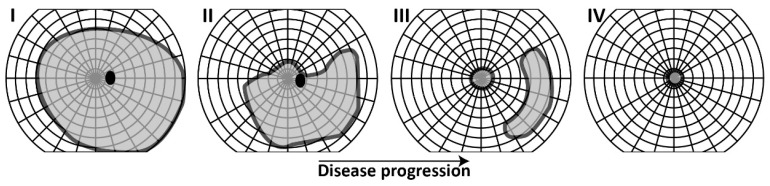
Illustrative example of typical visual field progression in a patient with retinitis pigmentosa using kinetic perimetry. Visual fields can be within normal limits in early stages of disease (**I**), although visual field defects may already be present but not detectable within the used target stimulus. With time, constriction of the visual fields occurs, with defects typically being symmetric and expanding more rapidly outwards and slower inwards (**II**,**III**). Ultimately, a small central remnant of visual field may remain in end-stage retinitis pigmentosa, which is commonly experienced and known as ‘tunnel vision’ (**IV**). Note that the clinical course of visual field loss varies between individuals and may follow a progression pattern that is different from this illustration.

RP involves the primary degeneration of rods, followed by the secondary degeneration of cones [2]. As each photoreceptor type plays a specific role in the establishment of vision, there is a classic order in which the clinical symptoms of RP manifest. Due to the initial loss of rod photoreceptors, which are primarily used for vision in dim light conditions and peripheral visual functions, patients experience difficulty or inability to see in dark or dimly lit environments, which is commonly known as ‘night blindness’ or nyctalopia [15]. The second symptom found in RP is a progressive loss of peripheral visual fields, although this may be unnoticed in the initial stages of disease due to compensating mechanisms [53]. When the degeneration of photoreceptors further expands, so do the visual field defects. Constriction of visual fields progresses over time, eventually reaching the central part of the visual field. In advanced stages of RP, only a small residual central island of visual field may remain—with or without peripheral remnants—which results in severely constricted vision known clinically as ‘tunnel vision’ (Figure 2) [54,55]. As a result of visual field loss, one of the major perceived difficulties in patients with RP is mobility, which requires input from both central and peripheral vision [56].

Cone photoreceptors, which are densely packed in the macula, are responsible for visual acuity and color vision [57]. Gene variants that target specifically rods but not cones (e.g., disease-associated variants in the *RHO* gene affecting rhodopsin, a rod-specific protein) can still cause death of cone photoreceptors. It remains unclear how cone degeneration in these specific circumstances occurs. Several theoretical concepts have been suggested for the secondary degeneration of cones, including the lack of trophic factors, such as rod-derived cone viability factor, nutrient shortages, oxidative stress and microglial activation, which are induced following rod photoreceptor apoptosis [57,58,59,60]. Loss of cone photoreceptors leads to a gradual loss of central vision once sufficient cones in the macula are compromised. This process can ultimately lead to severe visual impairment or even functional blindness based on criteria established by the World Health Organization [61]. Importantly, most patients with RP in advanced stages of their disease will likely retain some degree of residual vision, and total blindness, i.e., no light perception, is uncommon [62]. Previous studies reported that 7–8% of patients with generalized RP end up with a vision of counting fingers or worse in their fourth or fifth decade of life, while less than 1% of RP patients progress to no light perception [62,63]. In addition to central vision loss, patients may lose color vision, and they may have increased sensitivity to light (i.e., photophobia) [15,64]. Photopsia, i.e., seeing light flashes or static noise when no light enters the eye, is very common in RP, possibly due to reduced afferent nerve impulses or spontaneous signaling from the inner retina [1,65].

## 5. Disease Onset and Prognosis

The onset, severity and progression of symptoms in RP are highly variable, even in affected individuals from the same family. (Epi)genetic and possibly environmental modifiers are believed to contribute to phenotypic variability, which complicates the establishment of potential genotype–phenotype correlations [66]. It remains difficult to establish a visual prognosis for RP as a group of conditions, although a rough estimate of disease progression can be determined based on the mode of inheritance and the underlying genetic defect, as well as previous information on the clinical course [67]. More severe phenotypes with early-onset disease and the rapid decline in visual function are typically observed in patients with arRP or XLRP, as these variants generally result in loss of function of a crucial protein in the visual pathway [14,68,69,70,71]. High myopia (refractive error of −6 diopters or more) may be associated with a more rapid disease progression, for instance in *RPGR*-associated X-linked RP [69,71,72]. In contrast, patients with adRP (e.g., due to *RHO* mutations) mostly demonstrate a relatively mild disease course compared to arRP or XLRP, and they may even retain considerable central and peripheral visual function up until the eighth decade of life [73,74,75]. The disease course of RP is best understood in the most prevalent genes associated with RP (e.g., *RHO* and *RPGR*) as more extensive retrospective and prospective studies have been performed in these genes; thus, their visual prognosis can be more accurately estimated [23,68,71,73,74,76,77,78].

## 6. Diagnostic Testing in RP

The management of RP starts by establishing the diagnosis through extensive clinical and genetic testing. Early diagnosis of RP enables early prevention and management of complications, disease monitoring and genetic counselling (e.g., family planning). Clinical examination, including the assessment of visual functions, provides relevant information for visual rehabilitation services and helps affected individuals make informed choices about their professional life. Genetic testing is important for visual prognosis, family planning, and for potential inclusion into clinical trials and gene therapy when available. In this chapter, we discuss the principles of clinical and genetic testing methods used for the diagnosis of RP.

## 7. Clinical Testing and Evaluation

Clinical evaluation of patients with presumed RP consists of a comprehensive ophthalmic examination that includes best-corrected visual acuity (BCVA), intraocular pressure, slit-lamp, fundus, perimetric, retinal imaging, and electrophysiological evaluation.

### 7.1. Fundus Findings

The classical clinical hallmarks of RP seen in fundus examinations include a pale optic disc, retinal vessel attenuation and intraretinal hyperpigmentation. While intraretinal hyperpigmentation typically has a bone-spicule-like appearance, it may also present as nummular, salt and pepper-like, or with granular pigmentation A non-pigmented form of RP also exists (‘RP sine pigmenti’), instead of the typical bone-spicule-like hyperpigmentation [1,79,80]. These retinal changes typically occur bilaterally and show a high degree of symmetry, although cases of unilateral RP have also been described [81,82]. Other fundus findings, albeit less common, include optic nerve drusen, CME, epiretinal membrane formation, and Coats-like disease, a (mid)peripheral exudative vasculopathy characterized by telangiectatic vessels, focal serous retinal detachment and lipid exudate deposition [66]. The onset and presentation of the aforementioned fundus findings differ highly between individuals and may even present in atypical forms. Sector RP is considered an atypical, mild form of RP, which is more common in patients with adRP [69,73,83,84,85]. Degeneration in sector RP has a predilection for the inferior nasal hemisphere of the retina with corresponding superior visual field defects [58]. A widespread, generalized disease similar to classic RP may develop with time, although this is not necessarily the case for all patients with sector RP [73].

### 7.2. Differential Diagnosis

A complete medical history, review of other body systems and sometimes laboratory testing is necessary to distinguish between RP and other conditions that can masquerade as RP. The list of differential diagnoses in RP is extensive and includes infectious (e.g., syphilis or congenital rubella), drug-induced (e.g., chloroquine or thioridazine), iatrogenic (e.g., laser photocoagulation), metabolic (e.g., gyrate atrophy due to hyperornithinemia) and nutritional etiologies (e.g., vitamin A and zinc deficiencies), as well as a range of non-RP-inherited retinal dystrophies (e.g., choroideremia, congenital stationary night blindness and Oguchi disease) [15,86]. In addition, it is important to rule out several metabolic diseases that may present with fundus findings mimicking RP, including abetalipoproteinemia (Bassen–Kornzweig disease), ataxia with vitamin E deficiency and adult Refsum disease, among others [51,87,88]. This distinction from RP is crucial as disease progression in some metabolic diseases can be combated. For instance, in the case of Abetalipoproteinemia and ataxia with vitamin E deficiency, disease progression can be slowed with specific vitamin supplements, while disease progression in adult Refsum disease can be slowed by limiting the intake of food high in phytanic acid [89,90]. A delayed diagnosis and, consequentially, delayed treatment may have significant and irreversible consequences for patients with these diseases [88].

### 7.3. Electrophysiological Testing

**Figure 3:**
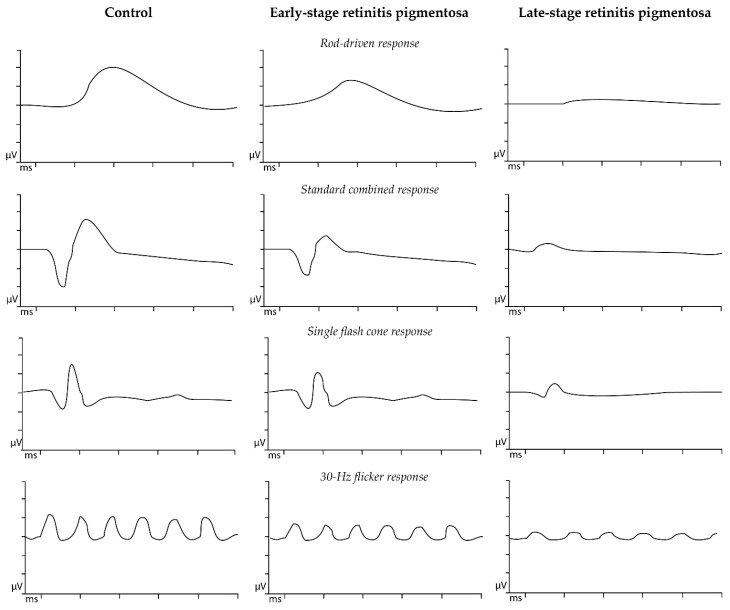
Example full-field electroretinography recordings in a healthy patient and in patients with different disease stages of retinitis pigmentosa. Different stimuli are used to establish the diagnosis of retinitis pigmentosa, which is based on the guidelines of the International Society for Clinical Electrophysiology of Vision (ISCEV). In patients with advanced stages of diseases, rod-driven responses are severely diminished or even absent, whereas residual cone-driven responses may still remain.

Electrophysiological testing plays a major role in the diagnosis and follow-up of RP, as well as the differentiation of RP from other diagnoses. Among all electrophysiological tools, full-field electroretinography (ffERG) is the most common technique used for diagnosing RP, which follows the guidelines established by the International Society for Clinical Electrophysiology of Vision (ISCEV) [91]. In brief, the ffERG evaluates the retinal function in response to light stimulus. A dim white single flash in a dark-adapted eye (i.e., scotopic test conditions) invokes a rod response, whereas a flickering white light (30-Hz) in a light-adapted eye elicits a cone response [91]. When RP becomes detectable in ffERG, i.e., when the retina is sufficiently affected, scotopic responses demonstrate a significant reduction in amplitudes of both a- and b-waves, which are responses mostly derived from photoreceptor and bipolar cells, respectively (Figure 3). Ultimately, both scotopic and photopic responses can be fully extinguished and are non-recordable in end-stage disease [15]. Other diagnostic tools that measure retinal function include multifocal ERG (mfERG), which assesses macular function, and dark adaptometry, which measures the time it takes for photoreceptors to retain maximal sensitivity following photoreceptor bleaching [92,93,94]. These other electrophysiological testing tools play a smaller role in the initial diagnosis of RP, and are instead sometimes used to complement ffERG/clinical findings and to rule out other potential diagnoses.

### 7.4. Perimetry Testing

As ffERG responses eventually become non-recordable in patients with advanced forms of RP, ffERG is not useful for monitoring disease progression [95]. Instead, kinetic visual fields and multimodal imaging techniques are used to further monitor progression, as these can be utilized even in advanced stages of disease.

Visual field testing is a key in the functional evaluation of RP. When performed in early phases of disease, visual field testing demonstrates progressive, midperipheral visual field loss. With time, a midperipheral ring scotoma develops, which typically expands more rapidly towards the periphery than centrally [66]. Goldmann perimetry is often considered the standard for the detection of visual field progression in RP. In Goldmann perimetry, a light stimulus is presented outwards and is slowly moved inwards by an operator until the stimuli are visibly seen by the patient [96]. This process is then repeated multiple times while using different stimuli, in order to map the extent of a patient’s visual field. Limitations of Goldmann kinetic perimetry include significant variability in patients with low vision/unstable fixation and inter-operator variability [97,98,99]. While Goldmann kinetic perimetry is still commonly used in clinical settings, it is gradually being replaced by other visual-field-testing methods, such as computerized (semi-)automated perimetry devices, in clinical practice, research and clinical trials [95,100].

Microperimetry (MP) is a semi-automated perimetry device that correlates stimuli presented to the central retina using fundus tracking [100]. The test is performed by having the patient fixate on a central point while different stimuli are presented at various locations on the retina. The patient’s ability to perceive the stimulus at each location is recorded and used to create a ‘retinal sensitivity map’. This yields a more precise point-by-point correlation and follow-up [100]. MP is often employed in clinical trials for IRDs in combination with traditional outcome measures (i.e., visual acuity and visual fields) [101,102,103]. Recent studies have shown that changes in retinal sensitivity can be detected within relatively short time frames, preceding changes in BCVA [95,104,105]. As BCVA is affected in later stages of RP, it is difficult to assess disease progression based on BCVA in short follow-up periods, such as in the context of clinical trials [103]. Therefore, MP can prove beneficial in clinical trials as a complementary outcome measure to detect disease progression and to assess treatment outcome. It is important to note that MP is not a replacement for traditional visual acuity testing as it is not appropriate for all patients with RP. Measuring disease progression with MP becomes more difficult in patients with poor fixation (e.g., patients with low vision or nystagmus), which in turn causes variability in measurements. Another limitation is that MP only allows for sensitivity mapping of the central retina.

Dark-adapted (DA) static perimetry was developed to measure rod-and-cone function across larger extents of the retina [106,107]. In contrast to light-adapted perimetry, DA can be used to discriminate between rod and cone functions by testing each loci with different stimuli [108]. Each testing loci is exposed to a cyan (505 nm) and red (626 nm) stimuli. As rods are less sensitive to red stimuli, a large threshold difference between stimuli indicates rod mediation [109]. DA static perimeters are commercially available but can also be performed on current standard perimeters by modifications [108].

### 7.5. Full-Field Stimulus Threshold Testing

Another psychophysical tool is the full-field stimulus threshold (FST), which has become a key outcome measure in gene-therapy trials [31,68,110]. The FST was developed as a tool to quantify retinal sensitivity in patients with end-stage IRD as these patients commonly lacked the vision and fixation needed for other outcome measurements tools [111]. In brief, the purpose of the FST is to measure the retinal threshold, which is defined as the stimulus intensity and is seen 50% of the time by a patient. Different stimuli (red, blue and white) yield differentiation between rod, cone or mixed rod–cone responses, and stimuli are typically presented multiple times to account for test–retest reliability. As the FST measures the thresholds of the entire retina, a limitation of this measurement tool is the lack of spatial information. Still, the FST has been able to demonstrate treatment efficacy across multiple gene-therapy trials [20,21,23,111,112].

### 7.6. Multimodal Imaging

**Figure 4:**
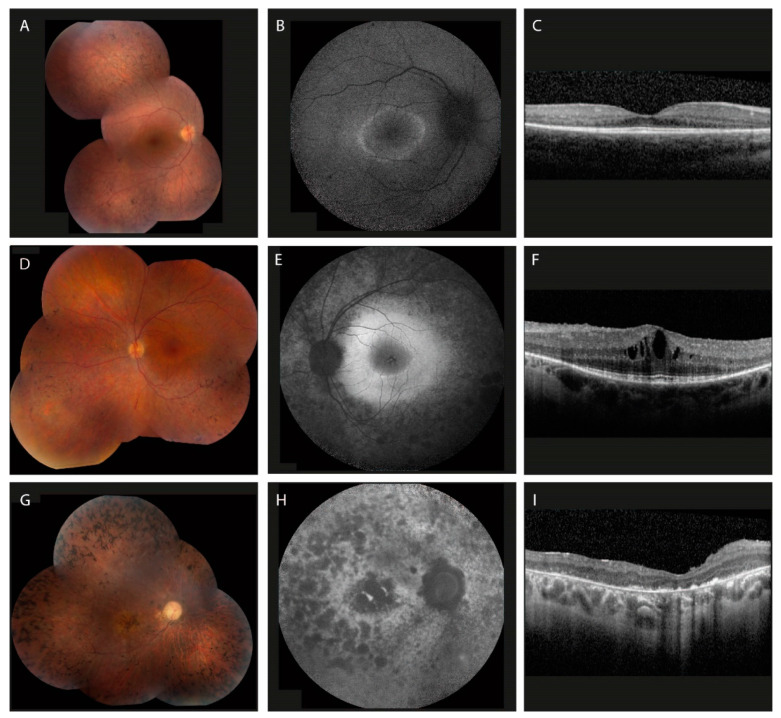
Multimodal imaging in three patients with retinitis pigmentosa (RP). (**A**–**C**): Multimodal imaging in a patient with RP caused by a variant in the *RHO* gene showing the clinical hallmarks of RP, including attenuated vessels and bone-spicule-like hyperpigmentation in the (mid)peripheral retina (**A**). On autofluorescence imaging, a small hyperfluorescent ring is observed in the macula (**B**). Spectral-domain optical coherence imaging shows a relatively intact central retina with loss of the outer retinal layers (i.e., ellipsoid zone and external limiting membrane) outside this area (**C**). (**D**–**F**): Multimodal imaging in a different patient with *RHO*-associated RP reveals hypo-autofluorescent areas in the midperipheral retina and around the vascular arcades, with a broad hyperautofluorescent ring-like region in the macula (**E**). The foveal area shows hypo-autofluorescence some petaloid, likely due to the presence of cystoid macular edema that masks underlying autofluorescence (**F**). SD-OCT confirms the presence of CME, along with the perifoveal loss of the outer retinal layers. (**G**–**I**): More extensive bone-spicule-like hyperpigmentation is observed in this patient with advanced *RPGR*-associated RP, showing not only hyperpigmentation in the midperipheral retina, but also in the fovea (**G**). Autofluorescence imaging (**H**) shows some residual regions of normal or increased autofluorescence, together with regions of mottled hypo-autofluorescence that also include the fovea. As expected, there is clear outer retinal and retinal pigment epithelium on optical coherence tomography (**I**).

Multimodal imaging, including widefield fundus imaging, spectral-domain optical coherence tomography (SD-OCT), and fundus autofluorescence (FAF) imaging, is used to visualize the extent of retinal degeneration in patients with RP. Widefield fundus imaging yields a comprehensive overview of the retina, which can be used to monitor progression in RP. Multiple studies have used structural markers on SD-OCT, such as the central retinal thickness and/or ellipsoid zone (EZ) band width, as another means of tracking disease progression [113,114,115,116,117,118,119]. In addition, SD-OCT yields the detection of secondary complications associated with RP, such as the presence of CME and epiretinal membrane. FAF is a non-invasive imaging technique that measures the level of autofluorescent lipofuscin components in the photoreceptors and RPE. A hyperautofluorescent macular ring can typically be observed in earlier disease stages of RP and indicates the transition zones between healthy and degenerating retina, which are often accompanied by progressive thinning of the EZ, external limiting membrane (ELM) and outer nuclear layer (ONL) on SD-OCT (Figure 4) [78]. It is important to note that hyperfluorescent rings are not specific to RP and can also be seen in other retinal diseases such as cone–rod dystrophies. Gradual constriction of hyperautofluorescent rings towards the central retina occurs in RP, whereas gradual expansion of the ring is observed in cone–rod dystrophies due to differences in order of photoreceptor degeneration. In advanced stages of RP, when extensive photoreceptor and RPE degeneration has occurred, resulting in the depletion of lipofuscin levels in the retina and RPE, extensive hypo-autofluorescent areas are seen on FAF (Figure 4).

## 8. Genetic Testing

Due to the clinical variability of RP and its phenotypic overlap with other IRDs, a diagnosis based on clinical findings alone is not sufficient. Therefore, genetic testing has become indispensable in the diagnosis and management of RP. With the approval of gene therapy for *RPE65*-associated IRD, and several first-in-human trials on other genetic therapies for a range of IRD-associated genes, it is pivotal to offer genetic testing to patients when available and affordable. Genetic testing allows for the assessment of a patient’s potential eligibility for these ongoing and upcoming trials and facilitates genetic counseling and provides a more accurate clinical prognosis [120]. There are several genetic diagnostic techniques available, and we briefly discuss the advantages and disadvantages of these modalities.

### 8.1. Sanger Sequencing

Sanger sequencing, a first-generation sequencing technique, has been the gold standard for DNA sequencing for several decades and is still considered by many to be the gold standard for single-gene or low-throughput sequencing [121]. Sanger sequencing starts with polymerase-chain-reaction amplification of the region of interest, followed by targeted sequencing of up to 800 base pairs [122,123,124]. While Sanger sequencing is fast and cost effective for single genes, it is outperformed by newer techniques when the sequencing of multiple targets is needed [125].

### 8.2. Next-Generation Sequencing

Next-generation sequencing (NGS), also called second-generation sequencing, is currently the primary approach for molecular analysis in IRDs. NGS distinguishes itself from Sanger sequencing by allowing for parallel sequencing of multiple parts of DNA from multiple samples (i.e., multiplexing). Because large amounts of DNA and RNA snippets can be sequenced in a short time using this method, it is also called high-throughput sequencing [126,127]. Currently, NGS can genetically solve up to 60–80% of all sequenced RP/IRD patients [128,129,130,131]. In the remaining unsolved patients, periodic re-examination of genomic data could prove valuable as new disease-causing variants are discovered and new bioinformatic and data analytical tools are developed over time. Within NGS, three main techniques exist that are used for the identification of genomic variants: targeted gene sequencing, whole-exome sequencing (WES) and whole-genome sequencing (WGS).

### 8.3. Targeted Gene Sequencing

Targeted gene sequencing allows for the sequencing of specific regions that are clinically relevant to the disease of interest. For RP, a custom gene panel is created that sequences all exonic and intronic regions associated with RP and related IRDs [122]. Targeted sequencing is an effective approach for initial screening of RP for several reasons as follows: it allows for greater read depth of targeted regions; regions are predefined and therefore more likely to be clinically relevant; and samples are screened at reduced costs and computational burden when compared to WES and WGS techniques [120]. Targeted gene sequencing is not useful for the detection of novel genes as these new regions are not sequenced until they are specifically added to the existing gene panel. If a novel gene is found for RP, previously used gene panels need to be redesigned and revalidated [120].

### 8.4. Whole-Exome Sequencing

WES exclusively targets protein-coding exons, also known as the exome, which makes up to approximately 1–2% of a patient’s entire genome [120,132]. WES provides coverage of more than 95% of the entire exome, in which 85% of all pathogenic variants are expected to reside [132]. Furthermore, WES can screen intronic variants close to target exons, e.g., splice-site variants [131]. As such, WES is a reliable tool to detect novel, mostly monogenic, variants in patients with genetically unsolved RP. A major limitation of WES is its inability to comprehensively detect structural variants, copy-number variants and chromosomal rearrangements [131].

### 8.5. Whole-Genome Sequencing

WGS targets the entire genome, which consists of over three billion nucleotides, and thus exceeds the coverage of previously mentioned NGS techniques [120]. This allows WGS to uncover variants not detected using WES, including copy-number variants, intergenic variants and deep intronic variants [120]. Despite the better coverage of WGS, there are several drawbacks that should be considered. Due to its wider coverage, WGS generates large clusters of information, more so than any other NGS technique, which includes an increase in secondary, accidental findings [133]. These large datasets obtained from WGS require greater levels of processing and analyzing, not to mention larger amounts of data storage and increased financial costs, compared to other NGS techniques [130,131].

### 8.6. Recommendations for Genetic Testing

In summary, considering the sheer number of genes involved in the pathogenesis of RP, NGS is often preferred over conventional Sanger sequencing. Out of all NGS techniques, targeted gene sequencing is typically the primary approach for genetic screening. Using broad, IRD-based gene panels allows for maximum coverage of relevant regions using a single test and provides the best balance between sensitivity, cost efficiency and computational burden compared to other NGS techniques [131]. When the underlying cause remains unresolved following targeted gene panel testing, other higher-targeting sequencing techniques (WES or WGS) can be employed to elucidate the exact genetic basis of the disease. Newer third-generation sequencing techniques also exist, which employ real-time DNA molecular sequencing and allow for longer reads [134,135]. However, these methods are still under development and are not commonly used in clinical practice.

## 9. Genetic Counseling

Because RP is a heritable disease, genetic counseling plays an important role in the management of RP. The aim of genetic counseling is to advise and inform patients of the physical, psychosocial and familial implications of genetic findings on RP [120,136]. Genetic counseling takes place prior to and after genetic testing and can be provided by a subspecialized ophthalmologist, clinical geneticist or by another specialized genetic counselor [40,136,137,138,139]. The organization of genetic counseling services differs between centers and across different countries, depending on the availability of genetic counseling professionals [120]. A recent study in the US demonstrated that most ophthalmologists (and/or optometrists) performed some degree of genetic counseling during patient visits, but these practices were often limited to taking a family history or explaining the inheritance pattern due to time constraints and/or due to limited knowledge in genetics [140]. Therefore, in most cases, patients should be referred to a clinical geneticist or genetic counselor for more comprehensive counseling. While both professions provide genetic counseling, clinical geneticists are physicians subspecializing in genetic testing, counseling and establishing the diagnosis, whereas genetic counselors primarily focus on providing counseling services [138].

Genetic counseling starts prior to genetic testing (i.e., pre-test counseling), in which patients are informed of the potential importance and implications of genetic testing for their disease, the limitations of genetic testing and potential ethical concerns [139,141]. Genetic counseling needs to be tailored to the needs and profile of the patient. Genetic counseling involves informing patients of the hereditary nature of their disease, the prognosis and management and the risk of the disease expressing itself in other family members [142]. Obtaining family data is important to determine the causality of newly discovered variants, for example, through pedigree mapping, linkage analysis and segregation analysis [143]. Recurrence risks are best estimated if the disease follows Mendelian inheritance laws and if the underlying genetic defect is known; thus, it is best discussed following genetic testing (i.e., post-test counseling). The diagnostic rates of genetic testing have improved due to the advent of NGS testing techniques, which have led to more personalized counseling and more accurate estimates of recurrence risks. However, these increased diagnostic rates have also led to an increase in incidental findings of variants of unknown significance. Genetic findings need to be correctly interpreted, placed into clinical contexts and appropriately conveyed to patients, which requires a high level of expertise on ophthalmogenetics [140].

With regard to genetic testing techniques, the likelihood of finding genetic mutations unrelated to the retinal disorder increases when techniques are able to detect more genetic variations [144,145]. These findings are known as secondary findings and are mostly found with WES and WGS [121]. This is an important aspect of counseling because patients also have the right “to not know”, which should be disclosed in the consent form for genetic testing [136,146,147]. Once a secondary finding is found, it may be ethically problematic to uphold this right to not know because a secondary finding can have implications for patient health or reproduction [137,148]. Each secondary finding should be assessed for their causality, clinical significance and actionability [149]. A list of recommended genes and variants has been published by the American College of Genetics and Genomics, which includes clinical significant genes, such as *BRCA1* and *BRCA2* [150]. Additionally, due to the lower read depth of WES and WGS (compared to more narrow techniques), there is a higher chance to miss a variant [124]. Another important aspect of genetic counseling is to psychologically guide patients who consider presymptomatic testing and to assess the social impact for the patient. For patients with RP, this may have an impact on informed choices about education, professional life and lifestyle. In some cases, diagnosis also has consequences for insurance, such as disability income insurance. If there is a higher risk of having affected offspring, then the option for preconception and pre-implantation counseling can and should be discussed.

### 9.1. Preconception Counseling

Once the mode of inheritance is established, genetic counselors are able to estimate the risk of recurrence and to counsel on reproductive choices. Several reproductive choices are as follows: (1) to conceive naturally—if the risk of inheritance is relatively low, the disease impact is judged acceptable, or if other options are in contrast with their personal beliefs; (2) to receive gamete or embryo donation—which allows for one parent to keep a genetic link with the child (via gamete donation), while also decreasing the risk of passing genetic conditions to their offspring; (3) to adopt—so that the genetic trait is not inherited, although the possibility for the adoptee to carry other medical health problems still remains; (4) or to decide to remain childless [120,151].

If patients decide to conceive naturally, it is also possible to screen whether the fetus is affected with an inherited eye condition, using prenatal testing if the causative genetic variants are known. Invasive prenatal genetic tests, such as chorionic villus sampling or amniocentesis, carry a small chance of miscarriage, which may deter patients from taking these tests, although this risk has been significantly reduced over recent decades [152]. Non-invasive prenatal testing (NIPT) also exists, which yields the detection of genetic conditions based on cell-free DNA in maternal blood, but this is not available yet for RP. A genetic counselor will be able to guide patients in selecting the right option for prenatal screening if required [153].

### 9.2. Pre-Implantation Genetic Testing

Another option for family planning is conceiving via assisted means, such as in vitro fertilization (IVF) or intracytoplasmic sperm injection (ICSI). Pre-implantation genetic testing (PGT) is then employed prior to IVF or ICSI, which is formerly known as pre-implantation genetic diagnosis [154,155]. PGT is a technique that screens the genetic material of an embryo after in vitro fertilization and before implantation [154,155,156,157]. In many ways, PGT resembles other forms of prenatal diagnostics. PGT can be subcategorized into six categories as follows: PGT-A (focused on aneuploidies screening); PGT-M (focused on monogenic disorders and diagnosing); PGT-SR (focused on structural rearrangements in a chromosome); combined PGT (combining PGT-A and PGT-M); extended PGT (focused on polygenic disorders); and non-invasive PGT (using blastocentesis or analysis of exhausted culture media as an alternative for embryo biopsy) [154,156]. PGT-M and combined PGT are mainly used to detect underlying gene variants linked to RP, while PGT-A and PGT-SR are subcategories describing screening focused on chromosome abnormalities. The subcategory also determines what kind of genetic screening method is used, with PGT-M mainly using NGS techniques [154]. The amount of DNA extracted for PGT-M testing is very low, thus pre-screening of the variants of interest is usually performed in order to increase the accuracy of the testing. This can be carried out by genetically testing both parents and possibly other family members, increasing the accuracy of detecting a single gene mutation [154]. The main advantage of PGT is the avoidance of selective abortion, as PGT makes it unlikely for the fetus to carry the screened genetic defect. Genetic counseling must always precede PGT, as patients must be informed of the advantages and limitations of this technique, and patients must understand that the possibility of misdiagnosis due to allele dropout, contamination or mosaicism is still present, although small [158,159].

## 10. Management of RP-Associated Complications

In the majority of patients, clinical management of RP remains symptomatic and is not curative in nature. There are several complications commonly found in association with RP, which should be closely monitored and, if possible, managed timely to minimize their impact. Below, we list several common and uncommon complications associated with RP, their potential impact on RP and suggested treatment options.

### 10.1. Cataract

Cataract is a common anterior segment complication in RP patients [160,161,162,163]. Cataract associated with RP is present at a younger age than those with age-related cataract, and most commonly is posterior subcapsular cataract (PSC), suggesting differences in the etiology of cataract formation between these two groups [161,162,164]. Previous studies have demonstrated that increased levels of pro-inflammatory cytokines and chemokines are present in the aqueous humor and vitreous fluid of patients with RP compared to the controls [164,165]. These increased inflammatory levels were mainly observed in younger patients and in those with significantly lower visual function, suggesting that a pro-inflammatory environment may play an important role in cataractogenesis in RP [164].

Significant cataract impairs visual function and additionally causes visual disturbances that may exacerbate existing functional symptoms in patients with RP [166,167,168]. The type of visual disturbances varies with the morphology of the lens opacity and includes symptoms of glare, photophobia and decreased contrast sensitivity, among others [166]. Straylight effects caused by cataract can aggravate visual disability [169,170]. Considering the impact of cataract in patients with RP, surgical removal of the lens opacity can be offered to improve visual function and to relieve any functional symptoms. Currently, the most used surgery technique for cataract removal is phacoemulsification of the natural lens and implantation of an artificial intraocular lens (IOL) [171,172]. In the absence of other (ocular) comorbidities, cataract surgery leads to significant improvements in visual function. However, in patients with RP, visual prognosis is less certain as the cause of progressive vision loss can be caused by the increased clouding of the lens, by the ongoing retinal degeneration by RP or a combination thereof. Patients with RP are also at increased risk for intra- and postoperative complications, including intraoperative phototoxic damage to the retina, (increase in existing) CM and zonular dialysis, among others [34,173,174,175,176,177]. Furthermore, higher rates of posterior capsular opacification and anterior capsule phimosis have been described following cataract surgery in patients with RP, which may also negatively influence visual outcomes if left untreated [178]. Despite the challenges regarding cataract surgery in RP, several studies have demonstrated its benefit, showing average BCVA improvements between studies (Table 1) [105,162,178,179,180,181,182,183,184,185,186]. Subjectively, visual improvement was reported in 44.8–96.7% of patients included in these studies [177]. Possible predictors for visual outcomes suggested by previous studies include the integrity of the EZ and ELM in the fovea and baseline BCVA [187,188,189]. Extensive loss of macular EZ integrity, often seen in patients with advanced stages of RP, may cause irreversible vision loss, leading to no or only modest visual gains after cataract surgery [179]. Some authors have advocated the use of low-light settings during surgery and the use of blue-light filtering IOLs in an attempt to limit additional retinal phototoxicity, although the evidence to support these preventive measures in RP is very limited [169,190].

The presence of new CME or the exacerbation of existing CME, with reported rates of up to 32% (Table 1), can negatively influence the visual outcome, and chronic CME may even aggravate photoreceptor loss in patients and thus should be timely managed [187]. For patients with RP, a previous study recommended the simultaneous postoperative use of topical nonsteroidal anti-inflammatory drugs and CAIs for at least 3 months to prevent the risk of CME [174]. Alternatively, or in addition, parabulbar steroids may be administered at the end of the operation in an attempt to reduce the likelihood of postoperative (increase in) CME. SD-OCT imaging in the pre- and postoperative care of patients with RP-associated cataracts is useful to monitoring CME.

Incidence rates of up to 13% of zonular dialysis following surgery have been reported. This increased risk of zonular dialysis is believed to be caused by a low-grade intraocular inflammation process in RP that causes weakened zonular attachments [173]. During preoperative intake, signs of zonular weakness can be present, including phacodonesis and lens subluxation, indicative of moderate to severe zonular weakness. However, zonular weakness is best observed while maneuvering the nucleus intraoperatively. Surgeons should avoid unnecessary manipulation and strain on the lens zonules by using optimal hydrodissection and bimanual rotation of the nucleus. Large capsulorrhexis can assist with optimal maneuvering, while also reducing the risk of capsular phimosis. The use of a capsular tension ring may also provide stability and decrease the risk of IOL (sub)luxation and anterior capsular phimosis, although the insertion itself of the capsular tension ring may also cause strain on the lens zonule system, so prophylactic insertion of such a ring may not be indicated [177,182]. IOL (sub)luxation at short- or long-term follow-up in RP has been reported in several case studies, and these cases were managed using scleral suture fixation or by replacing them with a range of anterior chamber IOLs after the (sub)luxated IOL had been removed, often requiring accompanying vitrectomy [191,192,193].

Posterior capsular opacification is another common complication after cataract surgery and is believed to develop faster in patients with RP, with a significant posterior capsular opacification occurring after a median time of 12–15 months postoperatively, reported by two studies [173,178]. It may already be pre-existent because of the presence of residual posterior capsular cataract remnants at the end of cataract surgery. Posterior capsular opacification can be treated with neodymium-doped yttrium-aluminum-garner laser capsulotomy, preferably using low energy levels, considering that this procedure can also induce CME [194].

In summary, the current literature suggests that cataract surgery is beneficial for a large group of patients with RP, provided that there is a good preoperative diagnostic evaluation, with postoperative improvements in both objective and subjective visual outcome measures [177]. Ophthalmologists are advised to employ SD-OCT imaging pre- and postoperatively to evaluate EZ integrity and CME, and to be aware of signs of zonular weakness. Patients should be counseled about the increased risk of complications and the guarded visual prognosis following surgery in order to set realistic expectations.

### 10.2. Cystoid Macular Edema

The presence of CME has been variably reported, with prevalence rates from 10% to up to 70% in at least one eye between different study populations [195,196,197]. While CME can occur in every genetic subtype of RP, it is more commonly found in patients with adRP [198]. Significant CME in RP may cause reduction in visual acuity, and if left untreated, it might cause further degenerative changes in the retina, including macular hole formation [199]. However, the short-term and long-term additional visual impact and detrimental influence of CME in RP has not been firmly established [196]. The advent of OCT imaging in clinical practice has made the detection of CME more efficient, allowing for earlier diagnosis and treatment. It should be noted that cystoid changes in patients with RP are not necessarily consistent with active fluid leakage and CME [67,200,201,202,203,204].

The specific pathophysiology of RP-CME remains to be elucidated, but multiple mechanisms have been proposed: leakage of fluid through the RPE due to insufficient RPE pumping fluid function; vitreomacular traction; breakdown of the blood–retina barrier; Müller cell dysfunction; and antiretinal antibodies [195,196]. Previous studies demonstrated that RP-CME typically resides within the inner nuclear layers (INLs) of the retina and does not cause significant disruption of the vascular plexus [205,206]. These findings support the hypothesis that RP-CME is more likely to be related to Müller cell dysfunction, rather than being vasculogenic, although other possible underlying mechanisms cannot be excluded to date. In the case of postoperative occurrence of CME, which occurs in up to 20% of RP patients after cataract extraction, there may be a more important role for a vasculogenic factor and active leakage [34,160,187].

Because the pathophysiology is not completely understood and different gene mutations are associated with different likelihoods of RP-CME, the appropriate treatment remains a subject of debate [207,208,209,210]. An in-depth review by Bakthavatchalam and colleagues on the treatment of RP-CME suggested that the oral carbonic anhydrase inhibitor (CAI) acetazolamide is an effective first-line treatment [197,209,210,211,212]. The exact mechanism of CAIs on RP-CME remains to be elucidated, but it has been postulated that CAIs selectively inhibit different carbonic anhydrase isozymes located in RPE cells, improving the polarity of RPE cells and improving fluid transport [213,214]. Several relatively small prospective and retrospective studies showed that oral intake of acetazolamide causes a significant reduction in central macular thickness in up to 80% of patients with CME [210,212,215]. While CAIs may restore retinal structure, its effect on retinal function, i.e., visual acuity, appears to be limited, and the long-term functional and anatomical benefit of reducing CME in RP remains to be observed [195,197,209,212,214,216]. In addition, there is a range of potential adverse effects of systemic CAIs, including paresthesia, malaise, nausea, altered taste, depression and drowsiness, as well as potential serum biochemical changes, including decreased serum potassium and increased chloride levels, thus discouraging prolonged use of CAIs [217,218]. In rare cases, patients also develop renal stones as a consequence of prolonged CAI use [219,220].

As an alternative to systemic CAIs, topical CAIs such as dorzolamide and brinzolamide can be used for the treatment of CME. Previous studies have shown a significant decrease in CME in 30–81% of study eyes following the use of topical CAIs, although the efficacy of systemic CAIs in reducing CME was higher than that of topical medication [197,209,215,221,222]. Topical CAIs can be prescribed if patients experience any adverse effect from systemic medication. Despite the significant reduction in CME, re-occurrence of CME after a period of discontinued use of CAIs is common [197,215,221,222,223,224,225,226]. Therefore, patients need to be actively monitored for recurrent CME, which requires restarting CAIs.

Furthermore, intravitreal injections with anti-vascular endothelial growth factor (anti-VEGF) have also been proposed as treatment for RP-CME [195,227]. Vascular endothelial growth factor (VEGF) is a protein important for angiogenesis, as well as for vasculogenesis [228,229]. Thus far, given the limited evidence of efficacy as well as the patient burden, there is no indication for anti-VEGF treatment for uncomplicated RP-CME.

Intravitreal injection of a dexamethasone implant has also been used for the treatment of RP-CME. A prospective study by Veritti and colleagues compared the efficacy of dexamethasone implants versus oral acetazolamide (30 eyes in each arm), demonstrating that dexamethasone implants caused more reduction in central macular thickness and a higher BCVA letter gain compared to oral acetazolamide [230]. While the use of dexamethasone implants for RP-CME may be promising, current evidence on its usage and long-term effects in RP-CME is limited [231,232,233]. Furthermore, intravitreal injections of dexamethasone implants can cause increased intraocular pressure, cataract formation and subconjunctival hemorrhages, as well as more severe and rare complications, such as retinal detachments, misplacement of the implant and endophthalmitis [234].

Based on the available literature, if there an indication for the treatment of RP-CME, CAIs are currently the preferred choice, with systemic CAIs preferred over topical CAIs because of their comparatively superior efficacy, provided that the profile of side effects are acceptable for the patient. Oral acetazolamide can be prescribed when there is significant central (fovea-involving) CME and patients should be informed of the common adverse effects, the possibility of refractory CME and the uncertainty regarding long-term benefit for visual function. More studies are needed on the long-term natural course of RP-CME, the use of anti-VEGF and steroid implants, the potential detrimental effect of cystoid fluid in the macula of RP patients and if treatment of CME has a short-term and long-term functional benefit.

### 10.3. Other Macular Abnormalities and Retinal Detachments

The prevalence of macular abnormalities, such as epiretinal membrane (ERM), macular hole and vitreomacular traction syndrome, has been estimated to be around 1.9% in patients with RP [235]. Significant epiretinal membranes cause visual disturbances (e.g., visual acuity loss, metamorphopsia and diplopia) and can also result in macular holes. The exact etiology behind epiretinal membrane formation remains unknown, although elevated inflammatory factors have been observed in the vitreous of patients with RP, suggesting that inflammation is likely a contributing factor [236]. Surgical outcomes for the treatment of the ERM in RP are limited; a study involving 10 RP patients that underwent pars plana vitrectomy and inner limiting membrane peeling for ERM showed improvements in retinal morphology for the majority of cases (82%), but no significant improvement in BCVA was observed [237].

Similarly, the occurrence of macular holes is rare in RP and, as a consequence, outcome rates of vitreoretinal surgery in patients with RP have only been reported in a select few case studies involving a small number of eyes [199,238,239,240]. The study by Jin and colleagues showed an improvement in visual acuity and structural integrity of the retina following pars plana vitrectomy in three out of five treated eyes, as well as an improvement in the sealing of the macular hole. The remaining patient, who also had extensive retinal detachment, showed no change in visual acuity [238]. A different case report by Garcia-Fernandez and colleagues showed that primary surgery resulted in closure of the macular hole in the treated patient, but reopening of the hole occurred after two years [240].

The prevalence of retinal detachments (RDs) in RP has been reported to be between 0.7% and 1.3% [241,242,243]. Retinal detachments occur at a relatively younger age in patients with RP than in those without RP. Retinal detachments are often rhegmatogenous in nature, although exudative and tractional forms have also been described [241]. In the study of Chan and colleagues, exudative RDs were mainly seen in patients with *CRB1*-associated IRDs [241]. In three previous studies, final reattachment rates between 86% and 96% were reported, using scleral buckling or vitrectomy, suggesting a favorable anatomical outcome [241,242,243]. An overview of surgical outcomes for retinal detachments in RP can be found in Supplementary Table S1.

### 10.4. Uveitis

Uveitis in patients with RP is relatively rare, with a prevalence estimated in one study at approximately 0.26%, although this is likely an underestimation as most patients have milder forms of uveitis and/or are asymptomatic [244]. Uveitis in RP most commonly presents as anterior uveitis, followed by intermediate uveitis and, even more rarely, as posterior uveitis [244,245,246,247]. Some forms of uveitis, such as acute zonal occult outer retinopathy and (atypical) advanced birdshot chorioretinopathy may mimic features of RP, such as pigment clumping and retinal vessel attenuation, which leads to initial misdiagnosis [248,249]. A specific form of uveitis found in patients with RP is Fuchs’ heterochromic uveitis, which has been reported in several case series [250,251,252,253,254,255,256]. The co-occurrence of uveitis in RP can be coincidental, but there may also be a role for underlying immunological abnormalities that play a role in the disease etiology of RP, which is supported by several animal and immunohistochemical studies [247,250,257,258].

Currently, there is limited evidence on the treatment of uveitis in RP. Only a few studies describe treatment modalities, and these case reports seem to show a low efficacy in preventing uveitis relapse [246,247]. Majumder and colleagues have described the use of topical, periocular and oral corticosteroids for the treatment of 22 patients with anterior and/or intermediate uveitis, with varying results. Two patients with anterior uveitis developed CME, which was resolved using topical nonsteroidal anti-inflammatory drugs. The management of uveitis did not show improvements in visual acuity at follow-up [244]. While the treatment of uveitis does not necessarily improve visual function, monitoring the activity of inflammation remains important to prevent further complications that may worsen visual function, such as CME formation and leakage of the optic nerve and/or retinal vessels, findings which have all been described in patients with RP [259,260,261].

### 10.5. Glaucoma

A common form of glaucoma found in RP is primary angle-closure glaucoma (PACG), with prevalence rates between 1.0% and 2.3% [262,263,264]. Previous studies have shown that the association between RP and PACG are related to nanophthalmos, short axial length, cataract and lens subluxation [263]. Anatomically, patients with a short axial length and/or cataract have a relatively shallow anterior chamber more prone to occlusion. Furthermore, the presence of zonular insufficiency and ectopia lentis in RP may cause forward displacement of the lens, which may also induce closing of the anterior chamber angle [264]. As PACG can cause irreversible optic nerve damage that may lead to further loss of remaining visual function in patients with RP, clinical work-up and timely intervention is crucial. In the acute setting, the overall goal for the management of PACG is to reduce intraocular pressure and to relieve angle closure. Glaucoma medications are given to lower intraocular pressure, to reduce pain and in preparation for laser peripheral iridotomy, which is the definitive treatment for PACG. Fellow eyes should also prophylactically receive an iridotomy as they are also at risk for developing PACG [265].

## 11. Rehabilitative and Psychological Management

The visual impairment caused by RP and the progressive nature of this disease may have detrimental effects on patients’ general health, self-sufficiency and independence, which can profoundly impact their own quality of life and that of their caretakers [266]. The impact of RP is diverse and may result in physical, mental, emotional and social disabilities. The extent to which the lives of patients are affected by RP varies greatly between individuals and relies on several factors, including their functional ability, age, daily activities, work, education, family, support networks and coping mechanisms [266]. Not all patients are aware of the rehabilitation services that can provide assistance for some of these aspects, and thus are left with unmet clinical needs [266]. Healthcare providers should screen patients for rehabilitation needs and, if desired, refer them to the appropriate services, such as low-vision rehabilitation, psychological counseling and mobility training services, which are commonly present in visual rehabilitation centers. The aim of these services is to help patients manage the consequences of their disease and to lead a lifestyle as autonomous as possible, optimizing their quality of life [267]. Low-vision rehabilitation services (LVRSs) encompass a multidisciplinary team that aims to achieve the maximum potential of a patient’s residual vision [268,269]. The composition of this multidisciplinary team varies between different countries and may include, but is not limited to, ophthalmologists, optometrists, occupational therapists, social workers and psychologists [267,270]. Multiple studies have demonstrated improvements in the quality of life in patients with visual impairment following LVRSs [271,272]. Rehabilitation services are tailored to a patient’s individual situation, which are based on a patient’s current visual abilities and their own rehabilitation goals [273]. Several instruments exist that can be used at the intake to screen for important rehabilitation needs, and to measure the efficacy of rehabilitation services. Common tools used at initial assessment within LVRSs may include variations of the National Eye Institute Visual Function Questionnaire, an instrument to measure vision-related quality of life, as well as the Activity Inventory, which systematically assesses the most important life domains and specific tasks for a patient [273,274,275]. A limitation of these aforementioned questionnaires is that they are not tailored to patients with RP, who may experience different difficulties than those, for example, with glaucoma. New questionnaires are being developed specifically for patients with IRDs in light of new upcoming therapies as a subjective outcome measure, such as the Michigan Retinal Degeneration Questionnaire [276].

Without rehabilitation, patients with visual impairment may have to abandon tasks, for instance, those that require detailed vision, such as reading [277]. A low-vision aid (LVA) yields improvement in visual performance and encompasses corrective glasses; filtering lenses; optical and non-optical LVAs (e.g., magnifiers, telescopes, reading stands); electronic assistive technologies, such as closed-circuit television, screen readers; and, more recently, portable electronic devices (e.g., Orcam or eSight) [278,279,280]. The efficacy of LVAs is demonstrated by improvements in reading speed and acuity in clinical studies, although knowledge on other important factors, such as the subjective preference and cost of LVAs, can also play an considerable role in the recommendation of these devices [277]. Simple adaptations can also be made at home, at school or at work to improve autonomous function and to create a safe environment [281]. Examples of these adjustments include improving lighting control, removing trip hazards and creating contrasts between objects for easier identification.

Blindness is often ranked as the worst medical condition by the general population among other very severe diseases, as well as being considered the medical condition with the highest impact on day-to-day life [282]. Nevertheless, the psychological consequences may be under-recognized. Loss of vision has been associated with depression, social isolation, sadness, anxiety and fear [283,284,285]. Few studies have investigated the psychological impact of LVRSs, which showed improvements in mental well-being following rehabilitation [286]. Further studies are needed to understand the effectiveness of LVRSs on mental health and whether the implementation of psychological interventions, such as cognitive behavioral therapy, should be routinely embedded in LVRSs [287].

For individuals with extensive visual field loss such as in RP, traveling independently can become increasingly difficult, especially in unfamiliar and poorly lit environments [288]. Many aspects of life are impeded by the inability to travel, such as social interaction and work; therefore, mobility impairment may also significantly impact an individual’s quality of life. In such cases, orientation and mobility training can be useful, which aims to teach patients to ambulate (un)known environments safely and independently. Examples of mobility training objectives include training on the use of a white cane when using public transport, riding a bike and using navigation devices while traveling [289].

LVRSs should be an integral part of the care for eye diseases, especially in patients with significant visual impairment, such as those caused by RP, to improve their independence and overall well-being. It is advisable to refer patients to LVRSs when unmet needs are evident, as well as when these needs are not so apparent, as low-vision centers provide many helpful services that are not necessarily known to a patient.

## 12. Investigational Treatment Modalities

Improved understanding of the underlying mechanisms of RP has driven current research, resulting in the dawn of novel treatment strategies. The timing and underlying mechanism causing retinal degeneration determines a patient’s eligibility for treatment. Below, we briefly explain the key features of current and emerging treatment modalities, their relevance in the treatment of RP and IRDs and their advantages and limitations.

### 12.1. Gene-Dependent Strategies

Ocular genetic therapies have become an emerging treatment modality for a wide variety of IRDs and have been successfully used in mice, dogs and now clinically in patients [18,23,24]. Retinal diseases appear to be excellent targets for gene-based therapies as the eye is highly compartmentalized, immune privileged, and are relatively accessible for local administration, while there is an elaborate armamentarium of structural and functional tests to evaluate treatment efficacy. Gene-based strategies are most effective in the early stages of disease as they aim to prevent further degeneration of the surviving target cells, whereas they are unable to restore cells that have already degenerated [1]. The term gene therapy encompasses different strategies based on the transfer and application to different nucleic acids.

### 12.2. Gene Augmentation Therapy

The most straightforward strategy is gene augmentation therapy, in which a wild-type (normal) copy of the mutant gene is delivered to the site of interest with the use of a vector in which the correct gene is packaged for delivery at the target cells. The vector that is generally used is an adeno-associated virus (AAV), which has been extensively researched, has high transduction efficiency and exhibits relatively low immunogenicity [290]. However, other viral and non-viral vectors are also studied, and each has its advantages and disadvantages [291]. The correct copy of the gene carried by the vector aims to compensate for the disease by restoring wild-type expression, thus preventing further disease. This method can be particularly useful for autosomal recessive and X-linked RP as these variants typically result in loss of function. In contrast, adRP may result in gain of function or dominant-negative variants, which may require alternative approaches, such as gene silencing or knockdown-and-replacements strategies [292]. In patients with *RPE65*-associated IRDs, subretinal administration of functional copies of *RPE65* using an adeno-associated virus vector resulted in functional improvements (e.g., BCVA, FST blue, and multi-luminance mobility test) [24,27,110,112,293]. A meta-analysis revealed that changes in BCVA were significant at 1 year after treatment, but afterwards declined to baseline BCVA 2–3 years post-treatment. It is possible that photoreceptors continue to degenerate due to insufficient delivery of functional genes, or that photoreceptors had already reached a pre-apoptotic state at the moment of therapeutic intervention [294]. A recent review demonstrated that the treatment effects of *RPE65* gene therapy lasts up to 7.5 years after administration, which suggests that multiple gene-therapy doses are needed to provide clinical stability during a patient’s lifetime [295]. A single dose of FDA-approved Luxturna costs approximately USD 425,000 per eye per treatment. Furthermore, a subset of *RPE65* patients developed chorioretinal atrophy as a side effect of the subretinal administration of gene therapy [296,297].

The challenges in gene augmentation strategies lie in the fact that it is a gene-specific therapy and thus cannot be universally applied for all IRDs. Each gene in RP varies in its clinical course, affected cell types and size, among other factors. Therefore, each gene may differ in its optimal timing for therapeutic intervention, the method of administration and its therapeutic delivery. While subretinal delivery has a more direct effect on photoreceptor cells, it provides treatment only for a limited region of the retina, thus requiring multiple or larger treatment zones for better outcomes [298]. Furthermore, intravitreal and subretinal delivery can induce immune and inflammatory responses, which can typically be managed with steroid therapy, but in rare cases may result in significant ocular inflammation with sight-threatening complications [299]. For many large genes in RP, such as *USH2A*, *ABCA4*, and *EYS*, AAV vectors cannot be used as a vehicle considering the limited packaging capacity of approximately 4.7 Kb [293,300]. Different viral vectors have been suggested, which differ in their gene-carrying capacity, cellular tropism, immunogenicity and mutagenicity [18]. Aside from *RPE65*, a range of RP-associated genes are currently targeted in gene-therapy trials, including but not limited to *RPGR*, *GUCY2D*, *XLRS*, and *CRB1* [18,294].

### 12.3. CRISPR/CAS9-Based Therapy

Gene editing strategies, such as repurposing the Clustered Regularly Interspaced Short Palindromic Repeats (CRISPR)-Cas9 system, have recently emerged as a potential solution for the limitations brought by gene augmentation strategies [293,294,301,302,303]. In CRISPR-Cas9 gene therapy, a Cas9 endonuclease is delivered to the target region via guide RNA, which causes double-strand breaks in the predefined regions of the genome. Subsequently, DNA-repair mechanisms are activated, namely non-homologous end joining (NHEJ) or homology-directed repair (HDR). Based on these two repair mechanisms, several types of gene editing can be performed. Using NHEJ, the ends of the cleaved DNA are ligated with or without the addition of base pairs, often resulting in gene inactivation. If multiple guide RNAs are introduced that target separates sites, NHEJ can be used to delete specific sequences. If a DNA template homologous to the target region is introduced alongside the CRISPR-Cas9 system, cells can even correct a gene, or insert a new gene using HDR mechanisms [301].

As with any form of gene therapy, the main challenges of CRISPR-Cas9 include the delivery of the CRISPR-Cas9 complex, and the potential risk of an immune response. In addition, a major drawback for the use of CRISPR-Cas9 therapies are potential off-target effects. When using the CRISPR-Cas9 system, the guide RNA may target different regions than intended due to similarities within the genome, subsequently resulting in unwanted genomic modifications [304]. Furthermore, HDR efficiency, which is required to correct IRD-causing variants, in retinal cells is low. HDR functions mainly in dividing cells and is not highly efficient in post-mitotic retinal cells [302].

### 12.4. Antisense Oligonucleotide Therapy

RNA therapies, such as antisense oligonucleotides (AONs), are an interesting treatment modality for IRDs, as they provide a possible solution for some patients with genetic variants not suited for AAV gene therapy, e.g., patients with splice-site defects [305,306]. AONs are short chains of nucleic acids that bind to a specific complementary messenger RNA (mRNA) to modify the expression of a given nucleotide sequence. The exact working mechanism differs between AONs, as they can be used, for example, to correct pre-mRNA splicing, for exon skipping or for mRNA knockdown [294].

There are some potential advantages of AONs over DNA-based therapies: AONs are relatively small in size and can fit current vectors; they do not directly modify DNA; and they do not induce double-strand breaks, thus not interfering with the endogenous expression of the target gene [307]. A limitation is that AONs have a limited duration effect based on their half-life and multiple intravitreal injections over the course of disease are likely needed [306]. Currently, no approved RNA therapies are available for IRDs and more data are needed to support the efficacy in this group of diseases, although several clinical trials are ongoing for variants in *CEP290*, *USH2A* and *RHO* [306].

## 13. Gene-Independent Strategies

### 13.1. Optogenetics

In late-stage RP, degeneration of photoreceptors may reach a point in which the window of therapeutic opportunity for ocular genetic therapies is surpassed. The remaining neurons, such as dormant cones and bipolar and retinal ganglion cells, are typically preserved until end-stage disease, making them possible targets for optogenetic therapies.

The key idea of optogenetic therapy is to deliver and express genetically encoded light-sensitive proteins called opsins to the remaining light-insensitive neurons in the inner retina of patients with RP via viral vectors [308]. Once opsins are expressed in these target cells, they can be stimulated by light and invoke a visual response, thus bypassing lost or damaged photoreceptors. If the targeted cells are connected to other cell types in the retinal circuit, light also modulates the activity of these cells. Optogenetic therapy can theoretically be applied to all patients with end-stage RP, regardless of genotype [309].

Several human clinical trials are ongoing that involve optogenetic therapy in patients with RP (NCT02556736, NCT03326336, NCT04919473, and NCT04278131). Different types of opsins have been used; however, all studies use an AAV2 or similar variant as a viral vector via intravitreal injections, targeting retinal ganglion or bipolar cells. In the study by Sahel and colleagues, partial recovery of visual function was observed in a patient with light perception vision that received the AAV vector containing the light-sensitive protein ChrimsonR. With light stimulation via engineered goggles, the patient was able to locate and perceive different objects in a controlled environment, demonstrating proof of concept for the use of optogenetic therapy in RP, although further optimization is likely needed [310].

### 13.2. Stem Cell Therapy

Stem cell therapy involves the use of stem cells to replace or repair cells in the retina and can be applied in patients with end-stage RP, regardless of genotype [302]. The treatment can be categorized by effect, i.e., the replacement or preservation of cells, and stem cell type as follows: embryonic stem cells (ESCs); induced pluripotent stem cells (iPSCs); hematopoietic stem cells; mesenchymal stem cells (MSCs); and retinal progenitor cells (RPCs) [311,312,313,314,315,316,317]. Stem cells with a higher cell potency, such as pluripotent ESCs and iPSCs, come with more extensive differentiation properties and can be used for the replacement of retinal cells [311]. These cells, as well as their derivatives, have a higher risk of tumorigenesis and uncontrollable cell migration when compared to lower-cell-potency stem cells [311,318]. The tumorigenesis of a treatment dose is closely monitored before administrating it to a patient, but no extensive long-term data are currently available [311]. RPCs can be derived from ESCs, iPSCs and MSCs, among others. These cells show promising results with increased BCVA outcomes in injected eyes but are relatively self-limiting regarding expansion compared to pluripotent cell lines [319,320]. RPCs also retain their capacity to differentiate in preclinical studies, which poses challenges post-transplantation [311,319,321]. MSCs, with their lower cell potency, are considered safer and have more long-term data on the risk of tumorigenesis. Patients treated with bone-marrow-derived MSCs showed initial improvements in BCVA, although their vision reverted to baseline at 12-month follow-up [313,314]. Stem cell therapy is still in the early stages of development, and further research is needed to refine and optimize its technique and to determine its safety and effectiveness in the treatment of IRDs. Important hurdles of stem cell therapy include potential immune rejection, tumorigenicity and surgical complications [321]. Nevertheless, it can be a promising treatment option for patients with end-stage retinal disease [313,322].

### 13.3. Retinal Prostheses

Electronic retinal implants are designed to provide a basic sense of visual function in severely visually impaired patients [294]. In essence, retinal prostheses stimulate remaining retinal neural cells with electrical pulses via an electrode array. This treatment is primarily intended for patients with little to no visual function as the current resolution of vision is low [323]. The number of electrodes, amount of stimulation and the remaining retinal function all play a role in the quality of perception created by retinal prostheses. Furthermore, patients require a relatively intact posterior visual pathway to ensure correct visual processing of light stimulation [323]. Retinal prostheses can be utilized via direct electrical stimulation, where an external processing unit (e.g., a digital camera mounted on eyeglasses) captures real-time images which are then transmitted to the retinal implant, or via photodiodes arrays, which are directly imbedded into the retinal space and convert projected light patterns into local electric currents.

Retinal implants can be installed in the epiretinal, subretinal or suprachoroidal space [324,325]. In epiretinal configuration, the implant is placed in the near vicinity and directly interacts with the retinal ganglion cells. In the subretinal configuration, the implant is positioned between the outer retinal layer and retinal pigment epithelium, at the site of the photoreceptors. The suprachoroidal approach was developed to prevent damage to the neural retina, as the stimulating electrode array is not directly attached to the retina. However, this meant that electrodes were placed further away from the intended cells, thus requiring higher currents for stimulation [324,326,327].

Several retinal implants have been developed, of which three have been regulatory-approved and implanted in over 500 patients over the past two decades as follows: Argus II, developed by Second Sight Medical Products, which was an epiretinal implant with glasses paired to a processing unit; ad Retina Implant Alpha-AMS and the Retina Implant Alpha-IMS by Retina Implant AG, which used a subretinal electrode array. Up to 20/1260 Snellen vision was achieved using Argus II, and 20/546 Snellen was achieved with the Retinal Implant Alpha-AMS [323].

The implants do not come without risks as up to 30–40% of Argus II users showed adverse events of conjunctival erosion, hypotony, conjunctival dehiscence or endophthalmitis within five months after implantation [328,329]. Alpha-IMS (by Retina Implant AG) showed increased intraocular pressure (IOP) caused by subretinal bleeding in 1 out of 19 patients (5.3%) [329]. Retina Implant AG and Second Sight Medical Products have withdrawn their current products, with the latter now testing a cortical visual prosthesis in an attempt to address a wider audience [323,330].

Retinal protheses are intended for patients with limited visual function, although the visual benefit with current techniques appears modest. Future developments in retinal prostheses should focus on increasing resolution of vision, visual fields and to minimize adverse effects as result of electrode array implantation, which require innovation from engineering, software and electrophysiological perspectives.

### 13.4. Neurotrophic Factors

Neurotrophic factors are proteins that promote the survival, differentiation and growth of neuronal cells. Several neurotrophic factors have been studied in animal models for the potential to treat retinitis pigmentosa, including ciliary neurotrophic, nerve growth, and brain-derived neurotrophic factors [331]. Improvements in scotopic and photopic responses were observed in eyes that received ciliary neurotrophic factor (CNTF) compared to control eyes. For clinical delivery, direct intravitreal or subretinal neurotrophic factor injections have been the most common route [331]. However, an implantable device has also been suggested as it allows for the long-term release of neurotrophic factors, minimizing the risk accompanied by repeated injections. Several clinical trials have been conducted to evaluate the safety and effectiveness of CNTF as a treatment for retinitis pigmentosa. In one phase 1/2 clinical trial, CNTF was administered to patients with retinitis pigmentosa via a slow-release implant in the eye. The results of this trial showed that CNTF was generally well tolerated and may have some beneficial effects on visual function in patients with retinitis pigmentosa [331]. Further randomized clinical trials evaluated the use of encapsulated-cell-ciliary neurotrophic factor implants for RP, showing no significant improvements in BCVA and visual field sensitivity for patients in the short (12 months) or long term (60–96 months) [332,333].

### 13.5. Neuroprotective Agents

In rod-specific retinal diseases, cone photoreceptors may still degenerate [49,50]. It is hypothesized that when large amounts of rods degenerate in RP, oxygen consumption in the retina is severely reduced, leading to the generation of large amounts of toxic free radicals [57]. These compounds are harmful to the remaining cone photoreceptors [2]. Additionally, the production of rod-derived cone viability factor is also affected, making cone receptors more vulnerable to degeneration [59,60]. N-acetylcysteine (NAC) and its more potent version, N-acetylcysteine amide (NACA), are powerful antioxidants that have shown to preserve cone function in animal models of RP [3,4]. In the FIGHT-RP1 study, the therapeutic benefit of daily intake of NAC was investigated, which showed improvements in visual function over the study period of 6 months [334]. These improvements diminished once patients discontinued the study medication. A retrospective study by the same group found similar neuroprotective features in the macula, as measured on microperimetry [334]. Another studied neuroprotective factor includes cerium oxide nanoparticles (CeO_2_-NPs), which are nanocrystals with antioxidative effects derived from the rare earth element cerium [335]. In rat models, these have been shown to be effective in preserving photoreceptor function, as well as slowing down the loss of photoreceptors [336,337]. So far, ophthalmological human clinical trials have not been conducted. Currently, no neurotrophic drugs have been regulatory approved.

### 13.6. Nutritional Therapies

Dietary supplements, such as vitamin A, lutein and docosahexaenoic acid (DHA) supplements, have been previously studied in patients with RP. Berson and colleagues published their study in 1993, where they assigned 601 non-genotyped RP patients with either 15,000 IU/d vitamin A, 15,000 IU/d vitamin A plus 400 IU/d vitamin E, trace amounts of both vitamins or 400 IU/d vitamin E [338]. The first two groups showed a slower decline in retinal function based on full-field cone electroretinography compared to the latter two. This group conducted a follow-up study in 2004, assigning RP patients with either DHA plus vitamin A (treatment group) or fatty acid plus vitamin A (control group), with a follow-up of two years. The authors concluded that the DHA + vitamin A group slowed the disease course of retinitis pigmentosa compared to patients in the group not assigned to DHA [339]. Similar effects of vitamin A supplements were also found in children by Berson and colleagues [340]. It has been postulated that because vitamin A is an important chromophore in the visual cycle, vitamin A supplementation can compensate for deficiencies in patients with RP [341]. Currently, less than 10% of the genes in RP involve genes associated with vitamin A metabolism [342].

A randomized clinical trial by Hoffman and colleagues (DHAX trial) investigated the use of high-dose DHA in patients with X-linked RP over the course of 4 years [343]. The results of this study demonstrated that DHA was not effective in slowing down rod or cone ERG progression. A second analysis of the DHAX trial revealed that DHA might reduce the rate of progression in final dark-adapted thresholds and visual field sensitivity parameters [343,344].

Recent reviews concluded that there was no clear benefit of vitamin A and/or DHA for patients with RP in terms of mean change in visual fields or ERGs [345,346,347]. An editorial by Massof and colleagues concluded that there was no convincing evidence that vitamin A is beneficial, and may even carry potential health risks [348]. Excess vitamin A compromises liver function and may cause birth defects [348]. Furthermore, careful consideration should be given to the possibility that RP is caused by specific genetic variants (e.g., in the *ABCA4* gene), as it has been shown in animal models that an excess of vitamin A may boost the accumulation of lipofuscin in the retina and accelerate disease progression [342,349,350].

Taken together, there is no strong evidence that supports the use of nutritional supplements for patients with RP. Nutritional supplements may slow down disease progression in IRDs closely tied to the vitamin A pathway in the retina (e.g., *LRAT*, *RPE65*, *RLBP1*, *RDH5*, and *RDH11*), although its clear benefit has not yet been sufficiently proven in studies [342]. Vitamin A should be avoided in patients with genetic subtypes susceptible for excess vitamin A (e.g., variants in *ABCA4*) as this may potentially accelerate disease progression [351]. Patients who do receive high doses of vitamin A should undergo laboratory work-up prior to therapy as longstanding use of vitamin A can result in toxicity (e.g., birth defects, liver failure, osteoporosis and central nervous system disorders) [142]. For these reasons, most ophthalmologists do not prescribe nutritional supplements to patients with RP as routine care.

## 14. Concluding Remarks

**Figure 5:**
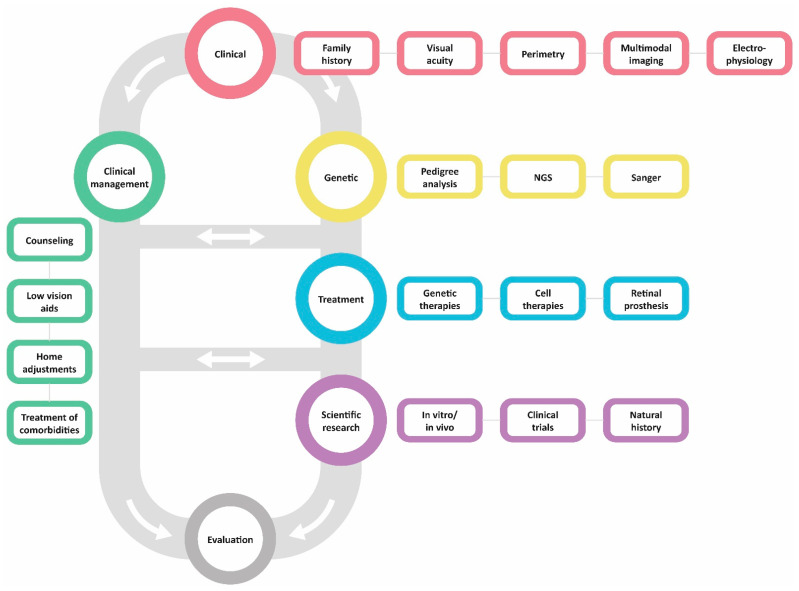
Flowchart demonstrating the clinical management of patients with retinitis pigmentosa (RP). The first step should be identifying patients with possible RP clinically, after which genetic testing should be performed, when available, if a diagnosis of presumed RP is made. Simultaneously, further clinical management should be offered through counseling, low-vision aids, home adjustments and treatment of comorbidities. Depending on the underlying causal gene, symptoms and severity of RP, treatment eligibility is assessed. Additionally, patients may opt to participate in ongoing research. The landscape for RP continues to change, and regular follow-up is advised to remain up to date with current clinical management and novel therapies.

The management of patients with RP is multidisciplinary and requires a focused and structured system where all healthcare providers involved in the care of patients with RP closely collaborate. Our increased understanding of the underlying disease mechanisms in RP have resulted in the development of novel treatment modalities, each with their own advantages and limitations. The treatment landscape in RP continues to evolve, and more research is needed to assess which treatment approaches are most beneficial to specific subgroups of patients with RP. Confirming the clinical and genetic diagnosis of patients with RP should be the first step in management, as many of the consecutive management steps rely on a thorough knowledge on the genetic and clinical characteristics. Disease monitoring, visual prognosis and enrollment of patients in upcoming and ongoing clinical trials are all steps that can be taken to further aid the patient. The evaluation and development of sophisticated, objective and subjective outcome parameters are needed to measure treatment efficacy of future clinical trials. With the era of NGS, the arrival of new diagnostic techniques has been one of the major milestones for unraveling the genetic background of RP and has aided the molecular detection of disease-associated genetic variants that could not be detected previously. A low threshold to refer to a genetic counselor is recommended as genetic counselors can interpret and translate the implications of genetic findings to patients with RP. Genetic counselors can facilitate informed reproductive decisions through preconception counseling and pre-implantation counseling, which can aid patients with family planning. The most common comorbidities found in RP, such as CME and cataract, can be managed using current treatment options. Coordination of visual rehabilitation between clinicians and low-vision rehabilitation centers optimizing patient outcomes and assists patients in performing daily life activities in order to maintain independence. Patients should be informed not only about new treatment developments, but also about currently available clinical management possibilities outside curative treatment, as they may provide relief of physical, psychological and social burden until early therapeutic intervention and prevention are possible. An example flowchart of the clinical management of RP is provided in Figure 5.

## Figures and Tables

**Table 1:** Overview of studies on cataract surgeries in patients with retinitis pigmentosa.

Study	Pts	Eyes	Follow-Up	Baseline BCVA	Postoperative BCVA	BCVA Change	Complications
Jackson et al., 2001 [160]	89	142	32.7 months	1.05 ± 0.38 logMAR	0.63 ± 0.49 logMAR	−0.42 logMAR	PCO (63%), CME (14%), CCS (10%)
Dikopf et al., 2013 [173]	47	80	23.3 months	1.23 ± 0.99 logMAR	0.81 ± 0.87 logMAR	−0.42 logMAR	PCO (83%), IOL dislocation (3%)
Bayyoud et al., 2013 [175]	52	46	26.0 months	1.45 ± 0.85 logMAR	1.32 ± 0.95 logMAR	−0.13 logMAR	PCO (44%), CME (4%), CCS (4%)
Garcia-Martin et al., 2013 [183]	35	35	1.0 month	0.10 ± 0.23 Snellen	0.48 ± 0.21 Snellen	0.38 Snellen	N/A
Nakamura et al., 2015 [188]	43	58	3.0 months	0.81 ± 0.51 logMAR	0.34 ± 0.43 logMAR	−0.47 logMAR	None
Yoshida et al., 2015 [162]	40	56	37.5 ± 22.6 months	0.76 ± 0.65 logMAR	0.45 ± 0.53 logMAR	−0.31 logMAR	PCO (84%), CCS (23%)
Davies et al., 2017 [174]	18	30	3.7 ± 3.3 months	1.09 ± 0.69 logMAR	0.61 ± 0.45 logMAR	−0.47 logMAR	CME (13.3%), PCO (66.7%)
Chan et al., 2017 [180]	42	67	6.9 ± 4.4 years	1.27 ± 0.42 logMAR	1.18 ± 0.49 logMAR	−0.09 logMAR	N/A
De Rojas et al., 2017 [179]	19	19	259 days	0.33 ± 0.20 logMAR	0.19 ± 0.17 logMAR	−0.14 logMAR	CME (32%), PCO (95%)
Lu et al., 2017 [186]	52	101	5.09 ± 2.2 months	0.12 ± 0.09 Snellen	0.21 ± 0.16 Snellen	0.09 Snellen	CCS (2%), increased IOP (2%)
Mao et al., 2018 [184]	70	109	3 months	0.80 ± 0.59 logMAR	0.45 ± 0.41 logMAR	−0.35 logMAR	N/A
Chatterjee et al., 2021 [185]	103	132	13.5 ± 25.1 months	1.21 ± 0.87 logMAR	0.66 ± 0.64 logMAR	−0.55 logMAR	PCO (17%), CME (5%), zonulolysis (3%). PCR (2%), uveitis (4%)
Chen et al., 2021 182]	63	84	6 months	1.3 ± 0.7 logMAR	0.91 ± 0.88 logMAR	−0.39 logMAR	CCS (5%)
Miura et al., 2021 [105]	62	62	3 months	0.45 ± 0.25 logMAR	0.11 ± 0.19 logMAR	−0.33 logMAR	None
Nakamura et al., 2022 [181]	64	96	5.8 ± 2.4 years	0.64 ± 0.52 logMAR	0.61 ± 0.52 logMAR	−0.03 logMAR	PCO (53%), CME (3%), ERM (2%), macular hole (1%), VMT (1%)
Nguyen et al., 2022 [178]	225	295	0.8 ± 1.6 years	1.03 ± 0.79 logMAR	0.81 ± 0.87 logMAR	−0.22 logMAR	PCO (38%), CME (5%), zonulolysis (5%), CCS (2%), IOL dislocation (1%), PCR (<1%), endophthalmitis (<1%)

BCVA = best-corrected visual acuity; PCO = posterior capsule opacification; CME = cystoid macular edema; CCS = capsular contraction syndrome; IOP = intraocular pressure; N/A = not available; PCR = posterior capsule rupture; Pts = number of patients; ERM = epiretinal membrane; VMT = vitreomacular traction.

## Data Availability

Not applicable.

## Supplementary Materials

The supporting information can be downloaded at: https://www.mdpi.com/article/10.3390/ijms24087481/s1.

## References

[1] Verbakel S.K., van Huet R.A.C., Boon C.J.F., den Hollander A.I., Collin R.W.J., Klaver C.C.W., Hoyng C.B., Roepman R., Klevering B.J. (2018). Non-syndromic retinitis pigmentosa. Prog. Retin. Eye Res..

[2] Hartong D.T., Berson E.L., Dryja T.P. (2006). Retinitis pigmentosa. Lancet.

[3] Haim M. (2002). Epidemiology of retinitis pigmentosa in Denmark. Acta Ophthalmol. Scand. Suppl..

[4] Na K.-H., Kim H.J., Kim K.H., Han S., Kim P., Hann H.J., Ahn H.S. (2017). Prevalence, Age at Diagnosis, Mortality, and Cause of Death in Retinitis Pigmentosa in Korea—A Nationwide Population-based Study. Am. J. Ophthalmol..

[5] Bunker C.H., Berson E.L., Bromley W.C., Hayes R.P., Roderick T.H. (1984). Prevalence of Retinitis Pigmentosa in Maine. Am. J. Ophthalmol..

[6] Xu L., Hu L., Ma K., Li J., Jonas J. (2006). Prevalence of retinitis pigmentosa in urban and rural adult Chinese: The Beijing Eye Study. Eur. J. Ophthalmol..

[7] Sen P., Bhargava A., George R., Ramesh S.V., Hemamalini A., Prema R., Ve R.S., Vijaya L. (2008). Prevalence of Retinitis Pigmentosa in South Indian Population Aged Above 40 Years. Ophthalmic Epidemiol..

[8] Nangia V., Jonas J.B., Khare A., Sinha A. (2012). Prevalence of retinitis pigmentosa in India: The Central India Eye and Medical Study. Acta Ophthalmol..

[9] Sharon D., Banin E. (2015). Nonsyndromic retinitis pigmentosa is highly prevalent in the Jerusalem region with a high frequency of founder mutations. Mol. Vis..

[10] Von Ammon F.A. (1838). Klinische Darstellungen der Krankheiten und Bildungsfehler des Menschlichen Auges, der Augenlider, und der Thränewerkzeug.

[11] van Trigt A.C. (1853). De Speculo Oculi. Nederlandsch Lancet.

[12] Langenbeck B.C.R. (1836). Observations Anatomico-Pathologica.

[13] Donders F.C. (1857). Beiträge zur pathologischen Anatomie des Auges. Graefe’s Arch. Clin. Exp. Ophthalmol..

[14] Musarella M.A., Macdonald I.M. (2011). Current Concepts in the Treatment of Retinitis Pigmentosa. J. Ophthalmol..

[15] Hamel C. (2006). Retinitis pigmentosa. Orphanet J. Rare Dis..

[16] Marmor M.F. (1979). The Electroretinogram in Retinitis Pigmentosa. Arch. Ophthalmol..

[17] Marmor M.F., Aguirre G., Arden G., Berson E., Birch D.G., Boughman J.A., Carr R., Chatrian G.E., Del Monte M., Dowling J. (1983). Retinitis Pigmentosa: A Symposium on Terminology and Methods of Examination. Ophthalmology.

[18] Aguirre G.D. (2017). Concepts and Strategies in Retinal Gene Therapy. Investig. Opthalmol. Vis. Sci..

[19] Chiu W., Lin T.-Y., Chang Y.-C., Lai H.I.-A.M., Lin S.-C., Ma C., Yarmishyn A., Lin S.-C., Chang K.-J., Chou Y.-B. (2021). An Update on Gene Therapy for Inherited Retinal Dystrophy: Experience in Leber Congenital Amaurosis Clinical Trials. Int. J. Mol. Sci..

[20] Maguire A.M., High K.A., Auricchio A., Wright J.F., Pierce E.A., Testa F., Mingozzi F., Bennicelli J.L., Ying G.-S., Rossi S. (2009). Age-dependent effects of RPE65 gene therapy for Leber’s congenital amaurosis: A phase 1 dose-escalation trial. Lancet.

[21] Bainbridge J.W., Smith A.J., Barker S.S., Robbie S., Henderson R., Balaggan K., Viswanathan A., Holder G.E., Stockman A., Tyler N. (2008). Effect of Gene Therapy on Visual Function in Leber’s Congenital Amaurosis. N. Engl. J. Med..

[22] Sodi A., Banfi S., Testa F., Della Corte M., Passerini I., Pelo E., Rossi S., Simonelli F., Italian IRD Working Group (2021). RPE65-associated inherited retinal diseases: Consensus recommendations for eligibility to gene therapy. Orphanet J. Rare Dis..

[23] Acland G.M., Aguirre G.D., Bennett J., Aleman T.S., Cideciyan A.V., Bennicelli J., Dejneka N.S., Pearce-Kelling S.E., Maguire A.M., Palczewski K. (2005). Long-Term Restoration of Rod and Cone Vision by Single Dose rAAV-Mediated Gene Transfer to the Retina in a Canine Model of Childhood Blindness. Mol. Ther..

[24] Acland G.M., Aguirre G.D., Ray J., Zhang Q., Aleman T.S., Cideciyan A.V., Pearce-Kelling S.E., Anand V., Zeng Y., Maguire A.M. (2001). Gene therapy restores vision in a canine model of childhood blindness. Nat. Genet..

[25] Ameri H. (2018). Prospect of retinal gene therapy following commercialization of voretigene neparvovec-rzyl for retinal dystrophy mediated by RPE65 mutation. J. Curr. Ophthalmol..

[26] Maguire A.M., Russell S., Wellman J.A., Chung D.C., Yu Z.-F., Tillman A., Wittes J., Pappas J., Elci O., Marshall K.A. (2019). Efficacy, Safety, and Durability of Voretigene Neparvovec-rzyl in RPE65 Mutation–Associated Inherited Retinal Dystrophy: Results of Phase 1 and 3 Trials. Ophthalmology.

[27] Pierce E.A., Bennett J. (2015). The Status of *RPE65* Gene Therapy Trials: Safety and Efficacy. Cold Spring Harb. Perspect. Med..

[28] Kapetanovic J.C., E McClements M., de la Camara C.M.-F., E MacLaren R. (2019). Molecular Strategies for RPGR Gene Therapy. Genes.

[29] Fischer M.D., McClements M.E., de la Camara C.M.-F., Bellingrath J.-S., Dauletbekov D., Ramsden S.C., Hickey D.G., Barnard A.R., MacLaren R.E. (2017). Codon-Optimized RPGR Improves Stability and Efficacy of AAV8 Gene Therapy in Two Mouse Models of X-Linked Retinitis Pigmentosa. Mol. Ther..

[30] Bennett J., Ashtari M., Wellman J., Marshall K.A., Cyckowski L.L., Chung D.C., McCague S., Pierce E.A., Chen Y., Bennicelli J.L. (2012). AAV2 Gene Therapy Readministration in Three Adults with Congenital Blindness. Sci. Transl. Med..

[31] Jacobson S.G., Cideciyan A.V., Roman A.J., Sumaroka A., Schwartz S.B., Heon E., Hauswirth W.W. (2015). Improvement and Decline in Vision with Gene Therapy in Childhood Blindness. N. Engl. J. Med..

[32] Ku C.A., Pennesi M.E. (2020). The new landscape of retinal gene therapy. Am. J. Med. Genet. Part C Semin. Med. Genet..

[33] Chivers M., Li N., Pan F., Wieffer H., Slowik R., Leartsakulpanitch J. (2021). The Burden of X-Linked Retinitis Pigmentosa on Patients and Society: A Narrative Literature Review. Clin. Outcomes Res..

[34] Antonio-Aguirre B., Swenor B., Canner J.K., Singh M.S. (2022). Risk of cystoid macular edema following cataract surgery in retinitis pigmentosa: An analysis of United States claims from 2010 to 2018. Ophthalmol. Retin..

[35] Bastek J.V., Heckenlively J.R., Straatsma B.R. (1982). Cataract Surgery in Retinitis Pigmentosa Patients. Ophthalmology.

[36] Chaumet-Riffaud A.E., Chaumet-Riffaud P., Cariou A., Devisme C., Audo I., Sahel J.-A., Mohand-Said S. (2017). Impact of Retinitis Pigmentosa on Quality of Life, Mental Health, and Employment Among Young Adults. Am. J. Ophthalmol..

[37] De Nadai K., Romano M.R., Binotto A., Costagliola C., Sato G., Parmeggiani F. (2011). Clinical and Rehabilitative Management of Retinitis Pigmentosa:Up-to-Date. Curr. Genom..

[38] Dryja T.P., Hahn L.B., Kajiwara K., Berson E.L. (1997). Dominant and digenic mutations in the peripherin/RDS and ROM1 genes in retinitis pigmentosa. Investig. Opthalmol. Vis. Sci..

[39] Daiger S., Rossiter BJ F., Greenberg J., Christoffels A., Hide W. (1998). Data services and software for identifying genes and mutations causing retinal degeneration. Investig. Ophthalmol. Vis. Sci..

[40] Branham K., Schlegel D., Fahim A.T., Jayasundera K.T. (2020). Genetic testing for inherited retinal degenerations: Triumphs and tribulations. Am. J. Med. Genet. Part C Semin. Med. Genet..

[41] Dias M.F., Joo K., Kemp J.A., Fialho S., Cunha A.D.S., Woo S.J., Kwon Y.J. (2018). Molecular genetics and emerging therapies for retinitis pigmentosa: Basic research and clinical perspectives. Prog. Retin. Eye Res..

[42] Jay M. (1982). On the heredity of retinitis pigmentosa. Br. J. Ophthalmol..

[43] Mathur P., Yang J. (2015). Usher syndrome: Hearing loss, retinal degeneration and associated abnormalities. Biochim. Biophys. Acta (BBA)-Mol. Basis Dis..

[44] Tatour Y., Ben-Yosef T. (2020). Syndromic Inherited Retinal Diseases: Genetic, Clinical and Diagnostic Aspects. Diagnostics.

[45] Blankman J.L., Long J.Z., Trauger S.A., Siuzdak G., Cravatt B.F. (2013). ABHD12 controls brain lysophosphatidylserine pathways that are deregulated in a murine model of the neurodegenerative disease PHARC. Proc. Natl. Acad. Sci. USA.

[46] Nguyen X.-T., Almushattat H., Strubbe I., Georgiou M., Li C.H.Z., van Schooneveld M.J., Joniau I., De Baere E., Florijn R.J., Bergen A.A. (2021). The Phenotypic Spectrum of Patients with PHARC Syndrome Due to Variants in *ABHD12*: An Ophthalmic Perspective. Genes.

[47] Yoshimura H., Hashimoto T., Murata T., Fukushima K., Sugaya A., Nishio S.-Y., Usami S.-I. (2015). Novel *ABHD12* Mutations in PHARC Patients. Ann. Otol. Rhinol. Laryngol..

[48] Jansen G.A., Oftnan R., Ferdinandusse S., Ijlst L., Muijsers A.O., Skjeldal O.H., Stokke O., Jakobs C., Besley G.T., Wraith J.E. (1997). Refsum disease is caused by mutations in the phytanoyl–CoA hydroxylase gene. Nat. Genet..

[49] Schwartz J.F., Rowland L.P., Eder H., Marks P.A., Osserman E.F., Hirschberg E., Anderson H. (1963). Bassen-Kornzweig Syndrome: Deficiency of Serum β-Lipoprotein: A Neuromuscular Disorder Resembling Friedreich’s Ataxia, Associated with Steatorrhea, Acanthocytosis, Retinitis Pigmentosa, and a Disorder of Lipid Metabolism. Arch. Neurol..

[50] Fiskerstrand T., Brahim D.H.-B., Johansson S., M’Zahem A., Haukanes B.I., Drouot N., Zimmermann J., Cole A.J., Vedeler C., Bredrup C. (2010). Mutations in ABHD12 Cause the Neurodegenerative Disease PHARC: An Inborn Error of Endocannabinoid Metabolism. Am. J. Hum. Genet..

[51] Waterham H.R., Wanders R.J.A., Leroy B.P., Adam M.P., Ardinger H.H., Pagon R.A., Wallace S.E., Bean L.J.H., Mirzaa G., Amemiya A. (1993). Adult Refsum Disease. GeneReviews^®^.

[52] Chen H.Y., Welby E., Li T., Swaroop A. (2019). Retinal disease in ciliopathies: Recent advances with a focus on stem cell-based therapies. Transl. Sci. Rare Dis..

[53] Weleber R.G., Gregory-Evans K., Ryan S.J., Hinton D.R., Schachat A.P., Wilkinson C.P. (2006). Chapter 17-Retinitis Pigmentosa and Allied Disorders. Retina.

[54] Grover S., Fishman G.A., Brown J. (1998). Patterns of visual field progression in patients with retinitis pigmentosa. Ophthalmology.

[55] Xu M., Zhai Y., MacDonald I.M. (2020). Visual Field Progression in Retinitis Pigmentosa. Investig. Opthalmol. Vis. Sci..

[56] Timmis M.A., Allsop J., Baranian M., Baker J., Basevitch I., Latham K., Pardhan S., Van Paridon K.N. (2017). Visual Search Behavior in Individuals With Retinitis Pigmentosa During Level Walking and Obstacle Crossing. Investig. Opthalmol. Vis. Sci..

[57] Campochiaro P.A., Mir T.A. (2018). The mechanism of cone cell death in Retinitis Pigmentosa. Prog. Retin. Eye Res..

[58] Georgiou M., Grewal P.S., Narayan A., Alser M., Ali N., Fujinami K., Webster A.R., Michaelides M. (2021). Sector Retinitis Pigmentosa: Extending the Molecular Genetics Basis and Elucidating the Natural History. Am. J. Ophthalmol..

[59] Aït-Ali N., Fridlich R., Millet-Puel G., Clérin E., Delalande F., Jaillard C., Blond F., Perrocheau L., Reichman S., Byrne L.C. (2015). Rod-Derived Cone Viability Factor Promotes Cone Survival by Stimulating Aerobic Glycolysis. Cell.

[60] Léveillard T., Sahel J.-A. (2010). Rod-Derived Cone Viability Factor for Treating Blinding Diseases: From Clinic to Redox Signaling. Sci. Transl. Med..

[61] World Health Organization International Classification of Diseases 11th Revision. https://icd.who.int/en.

[62] Vezinaw C.M., Fishman G.A., McAnany J.J. (2020). Visual impairment in retinitis pigmentosa. Retina.

[63] Fishman G.A. (1978). Retinitis Pigmentosa. Arch. Ophthalmol..

[64] A Fishman G., Young R.S., Vasquez V., Lourenço P. (1981). Color vision defects in retinitis pigmentosa. Ann. Ophthalmol..

[65] Bittner A.K., Diener-West M., Dagnelie G. (2009). A survey of photopsias in self-reported retinitis pigmentosa. Retina.

[66] Chang S., Vaccarella L., Olatunji S., Cebulla C., Christoforidis J. (2011). Diagnostic Challenges in Retinitis Pigmentosa: Genotypic Multiplicity and Phenotypic Variability. Curr. Genom..

[67] Fishman G.A., Fishman M., Maggiano J. (1977). Macular Lesions Associated With Retinitis Pigmentosa. Arch. Ophthalmol..

[68] Cideciyan A.V., Charng J., Roman A.J., Sheplock R., Garafalo A.V., Heon E., Jacobson S.G. (2018). Progression in X-linked Retinitis Pigmentosa Due to *ORF15-RPGR* Mutations: Assessment of Localized Vision Changes Over 2 Years rod and cone sensitivity in XLRP. Investig. Opthalmol. Vis. Sci..

[69] Nguyen X.-T., Talib M., van Schooneveld M.J., Brinks J., Brink J.T., Florijn R.J., Wijnholds J., Verdijk R.M., Bergen A.A., Boon C.J. (2020). RPGR-Associated Dystrophies: Clinical, Genetic, and Histopathological Features. Int. J. Mol. Sci..

[70] Sandberg M.A., Rosner B., Weigel-DiFranco C., Dryja T.P., Berson E.L. (2007). Disease Course of Patients with X-linked Retinitis Pigmentosa due to *RPGR* Gene Mutations. Investig. Opthalmol. Vis. Sci..

[71] Talib M., van Schooneveld M.J., Thiadens A.A., Fiocco M., Wijnholds J., Florijn R.J., Schalij-Delfos N.E., van Genderen M.M., Putter H., Cremers F.P.M. (2019). Clinical and Genetic characteristics of Male patients with RPGR-associated Retinal Dystrophies: A long-Term Follow-up Study. Retina.

[72] Talib M., van Schooneveld M.J., Van Cauwenbergh C., Wijnholds J., Brink J.B.T., Florijn R.J., Schalij-Delfos N.E., Dagnelie G., van Genderen M.M., De Baere E. (2018). The Spectrum of Structural and Functional Abnormalities in Female Carriers of Pathogenic Variants in the *RPGR* Gene. Investig. Opthalmol. Vis. Sci..

[73] Nguyen X.-T.-A., Talib M., van Cauwenbergh C., van Schooneveld M.J., Fiocco M., Wijnholds J., ten Brink J.B., Florijn R.J., Schalij-Delfos N.E., Dagnelie G. (2021). CLINICAL CHARACTERISTICS AND NATURAL HISTORY OF RHO-ASSOCIATED RETINITIS PIGMENTOSA: A Long-Term Follow-Up Study. RETINA.

[74] Berson E.L., Rosner B., Weigel-DiFranco C., Dryja T.P., A Sandberg M. (2002). Disease progression in patients with dominant retinitis pigmentosa and rhodopsin mutations. Investig. Opthalmol. Vis. Sci..

[75] Berson E.L., Sandberg M.A., Rosner B., Birch D.G., Hanson A.H. (1985). Natural Course of Retinitis Pigmentosa Over a Three-Year Interval. Am. J. Ophthalmol..

[76] Talib M., van Schooneveld M.J., van Genderen M.M., Wijnholds J., Florijn R.J., Brink J.B.T., Schalij-Delfos N.E., Dagnelie G., Cremers F.P., Wolterbeek R. (2017). Genotypic and Phenotypic Characteristics of CRB1-Associated Retinal Dystrophies. Ophthalmology.

[77] Talib M., Van Cauwenbergh C., De Zaeytijd J., Van Wynsberghe D., De Baere E., Boon C.J.F., Leroy B.P. (2022). *CRB1*-associated retinal dystrophies in a Belgian cohort: Genetic characteristics and long-term clinical follow-up. Br. J. Ophthalmol..

[78] Tee J.J., Yang Y., Kalitzeos A., Webster A., Bainbridge J., Michaelides M. (2019). Natural History Study of Retinal Structure, Progression, and Symmetry Using Ellipzoid Zone Metrics in RPGR-Associated Retinopathy. Am. J. Ophthalmol..

[79] Pearlman J.T., Flood S.R., Seiff S.R. (1976). Retinitis Pigmentosa Without Pigment. Am. J. Ophthalmol..

[80] Pearlman J.T. (1976). Letter: Nonpigmented Retinitis Pigmentosa and the Neurologist. Arch. Neurol..

[81] Weller J.M., Michelson G., Juenemann A.G. (2014). Unilateral retinitis pigmentosa: 30 years follow-up. BMJ Case Rep..

[82] Marsiglia M., Duncker T., Peiretti E., Brodie S.E., Tsang S.H. (2012). Unilateral Retinitis Pigmentosa: A Proposal of Genetic Pathogenic Mechanisms. Eur. J. Ophthalmol..

[83] Kranich H., Bartkowski S., Denton M.J., Krey S., Dickinson P., Duvigneau C., Gal A. (1993). Autosomal dominant ‘sector’ retinitis pigmentosa due to a point mutation predicting an Asn-15-Ser substitution of rhodopsin. Hum. Mol. Genet..

[84] Ramon E., Cordomí A., Aguilà M., Srinivasan S., Dong X., Moore A.T., Webster A.R., Cheetham M., Garriga P. (2014). Differential Light-induced Responses in Sectorial Inherited Retinal Degeneration. J. Biol. Chem..

[85] Shah S.P., Wong F., Sharp D.M., Vincent A.L. (2014). A Novel Rhodopsin Point Mutation, Proline-170-histidine, Associated with Sectoral Retinitis Pigmentosa. Ophthalmic Genet..

[86] Balfoort B.M., Buijs M.J., Asbroek A.L.T., Bergen A.A., Boon C.J., Ferreira E.A., Houtkooper R.H., Wagenmakers M.A., Wanders R.J., Waterham H.R. (2021). A review of treatment modalities in gyrate atrophy of the choroid and retina (GACR). Mol. Genet. Metab..

[87] Benomar A., Yahyaoui M., Meggouh F., Bouhouche A., Boutchich M., Bouslam N., Zaim A., Schmitt M., Belaidi H., Ouazzani R. (2002). Clinical comparison between AVED patients with 744 del A mutation and Friedreich ataxia with GAA expansion in 15 Moroccan families. J. Neurol. Sci..

[88] Jayaram H., Downes S.M. (2008). Midlife diagnosis of Refsum Disease in siblings with Retinitis Pigmentosa-the footprint is the clue: A case report. J. Med. Case Rep..

[89] Gouras P., E Carr R., Gunkel R.D. (1971). Retinitis pigmentosa in abetalipoproteinemia: Effects of vitamin A. Investig. Ophthalmol..

[90] Iwasa K., Shima K., Komai K., Nishida Y., Yokota T., Yamada M. (2014). Retinitis pigmentosa and macular degeneration in a patient with ataxia with isolated vitamin E deficiency with a novel c.717 del C mutation in the TTPA gene. J. Neurol. Sci..

[91] McCulloch D.L., Marmor M.F., Brigell M.G., Hamilton R., Holder G.E., Tzekov R., Bach M. (2015). ISCEV Standard for full-field clinical electroretinography (2015 update). Doc. Ophthalmol..

[92] Hood D.C., Odel J.G., Chen C.S., Winn B.J. (2003). The Multifocal Electroretinogram. J. Neuro-Ophthalmol..

[93] Alexander K.R., A Fishman G. (1984). Prolonged rod dark adaptation in retinitis pigmentosa. Br. J. Ophthalmol..

[94] Stavrou P., A Good P., Broadhurst E.J., Bundey S., Fielder A.R., Crews S.J. (1996). ERG and EOG abnormalities in carriers of X-linked retinitis pigmentosa. Eye.

[95] Menghini M., Cehajic-Kapetanovic J., MacLaren R.E. (2020). Monitoring progression of retinitis pigmentosa: Current recommendations and recent advances. Expert Opin. Orphan Drugs.

[96] Talib M., Dagnelie G., Boon C.J.F. (2018). Recording and Analysis of Goldmann Kinetic Visual Fields. Methods Mol. Biol..

[97] Barnes C.S., Schuchard R.A., Birch D.G., Dagnelie G., Wood L., Koenekoop R.K., Bittner A.K. (2019). Reliability of Semiautomated Kinetic Perimetry (SKP) and Goldmann Kinetic Perimetry in Children and Adults With Retinal Dystrophies. Transl. Vis. Sci. Technol..

[98] Barry M.P., Bittner A., Yang L., Marcus R., Iftikhar M.H., Dagnelie G. (2016). Variability and Errors of Manually Digitized Goldmann Visual Fields. Optom. Vis. Sci..

[99] Bittner A.K., Iftikhar M.H., Dagnelie G. (2011). Test-Retest, Within-Visit Variability of Goldmann Visual Fields in Retinitis Pigmentosa. Investig. Opthalmol. Vis. Sci..

[100] Pfau M., Jolly J.K., Wu Z., Denniss J., Lad E.M., Guymer R.H., Fleckenstein M., Holz F.G., Schmitz-Valckenberg S. (2021). Fundus-controlled perimetry (microperimetry): Application as outcome measure in clinical trials. Prog. Retin. Eye Res..

[101] Dimopoulos I.S., Tseng C., Macdonald I.M. (2016). Microperimetry as an Outcome Measure in Choroideremia Trials: Reproducibility and Beyond. Investig. Opthalmol. Vis. Sci..

[102] Schönbach E.M., Wolfson Y., Strauss R.W., Ibrahim M.A., Kong X., Muñoz B., Birch D.G., Cideciyan A.V., Hahn G.-A., Nittala M. (2017). Macular Sensitivity Measured With Microperimetry in Stargardt Disease in the Progression of Atrophy Secondary to Stargardt Disease (ProgStar) Study. JAMA Ophthalmol..

[103] Iftikhar M., Kherani S., Kaur R., Lemus M., Nefalar A., Usmani B., Junaid N., Campochiaro P.A., Scholl H.P., Shah S.M. (2018). Progression of Retinitis Pigmentosa as Measured on Microperimetry: The PREP-1 Study. Ophthalmol. Retin..

[104] Nguyen X.-T., Talib M., van Schooneveld M.J., Wijnholds J., van Genderen M.M., Schalij-Delfos N.E., Klaver C.C., Talsma H.E., Fiocco M., Florijn R.J. (2022). CRB1-Associated Retinal Dystrophies: A Prospective Natural History Study in Anticipation of Future Clinical Trials. Am. J. Ophthalmol..

[105] Miura G., Baba T., Tatsumi T., Yokouchi H., Yamamoto S. (2021). The Impact of Cataract Surgery on Contrast Visual Acuity and Retinal Sensitivity in Patients with Retinitis Pigmentosa. J. Ophthalmol..

[106] Bennett L.D., Klein M., Locke K.G., Kiser K., Birch D.G. (2017). Dark-Adapted Chromatic Perimetry for Measuring Rod Visual Fields in Patients with Retinitis Pigmentosa. Transl. Vis. Sci. Technol..

[107] Jacobson S.G., Voigt W.J., Parel J.-M., Apathy P.P., Nghiem-Phu L., Myers S.W., Patella V.M. (1986). Automated Light- and Dark- Adapted Perimetry for Evaluating Retinitis Pigmentosa. Ophthalmology.

[108] McGuigan D., Roman A.J., Cideciyan A.V., Matsui R., Gruzensky M.L., Sheplock R., Jacobson S. (2016). Automated Light- and Dark-Adapted Perimetry for Evaluating Retinitis Pigmentosa: Filling a Need to Accommodate Multicenter Clinical Trials. Investig. Opthalmol. Vis. Sci..

[109] Grewal M.K., Chandra S., Bird A., Jeffery G., Sivaprasad S. (2021). Scotopic thresholds on dark-adapted chromatic perimetry in healthy aging and age-related macular degeneration. Sci. Rep..

[110] Cideciyan A.V., Jacobson S.G., Beltran W.A., Sumaroka A., Swider M., Iwabe S., Roman A.J., Olivares M.B., Schwartz S.B., Komáromy A.M. (2013). Human retinal gene therapy for Leber congenital amaurosis shows advancing retinal degeneration despite enduring visual improvement. Proc. Natl. Acad. Sci. USA.

[111] Roman A.J., Cideciyan A.V., Wu V., Garafalo A.V., Jacobson S.G. (2022). Full-field stimulus testing: Role in the clinic and as an outcome measure in clinical trials of severe childhood retinal disease. Prog. Retin. Eye Res..

[112] Wang X., Yu C., Tzekov R., Zhu Y., Li W. (2020). The effect of human gene therapy for RPE65-associated Leber’s congenital amaurosis on visual function: A systematic review and meta-analysis. Orphanet J. Rare Dis..

[113] Cabral T., Sengillo J.D., Duong J.K., Justus S., Boudreault K., Schuerch K., Belfort Jr R.B., Mahajan V.B., Sparrow J.R., Tsang S.H. (2017). Retrospective Analysis of Structural Disease Progression in Retinitis Pigmentosa Utilizing Multimodal Imaging. Sci. Rep..

[114] Cai C.X., Locke K.G., Ramachandran R., Birch D.G., Hood D.C. (2014). A Comparison of Progressive Loss of the Ellipsoid Zone (EZ) Band in Autosomal Dominant and X-Linked Retinitis Pigmentosa. Investig. Opthalmol. Vis. Sci..

[115] Hariri A.H., Zhang H.Y., Ho A., Francis P., Weleber R.G., Birch D.G., Ferris F., Sadda S.R. (2016). Trial of Oral Valproic Acid for Retinitis Pigmentosa Group Quantification of Ellipsoid Zone Changes in Retinitis Pigmentosa Using en Face Spectral Domain-Optical Coherence Tomography. JAMA Ophthalmol..

[116] Liu G., Li H., Liu X., Xu D., Wang F. (2016). Structural analysis of retinal photoreceptor ellipsoid zone and postreceptor retinal layer associated with visual acuity in patients with retinitis pigmentosa by ganglion cell analysis combined with OCT imaging. Medicine.

[117] Phadikar P., Saxena S., Ruia S., Lai T.Y.Y., Meyer C.H., Eliott D. (2017). The potential of spectral domain optical coherence tomography imaging based retinal biomarkers. Int. J. Retin. Vitr..

[118] Schuerch K., Marsiglia M., Lee W., Tsang S.H., Sparrow J.R. (2016). Multimodal imaging of disease-associated pigmentary changes in retinitis pigmentosa. Retina.

[119] Xue K., Oldani M., Jolly J., Edwards T., Groppe M., Downes S.M., MacLaren R. (2016). Correlation of Optical Coherence Tomography and Autofluorescence in the Outer Retina and Choroid of Patients With Choroideremia. Investig. Opthalmol. Vis. Sci..

[120] Méjécase C., Malka S., Guan Z., Slater A., Arno G., Moosajee M. (2020). Practical guide to genetic screening for inherited eye diseases. Ther. Adv. Ophthalmol..

[121] Salmaninejad A., Motaee J., Farjami M., Alimardani M., Esmaeilie A., Pasdar A. (2019). Next-generation sequencing and its application in diagnosis of retinitis pigmentosa. Ophthalmic Genet..

[122] Kumar K.R., Cowley M.J., Davis R.L. (2019). Next-Generation Sequencing and Emerging Technologies. Semin. Thromb. Hemost..

[123] Crossley B.M., Bai J., Glaser A., Maes R., Porter E., Killian M.L., Clement T., Toohey-Kurth K. (2020). Guidelines for Sanger sequencing and molecular assay monitoring. J. Veter-Diagn. Investig..

[124] Ng P.C., Kirkness E.F. (2010). Whole genome sequencing. Methods Mol. Biol..

[125] Fahim A.T., Daiger S.P., Weleber R.G., Adam M.P., Ardinger H.H., Pagon R.A., Wallace S.E., Bean L.J.H., Mirzaa G., Amemiya A. (1993). Nonsyndromic Retinitis Pigmentosa Overview. GeneReviews®.

[126] Poptsova M.S., Il’Icheva I.A., Nechipurenko D.Y., Panchenko L.A., Khodikov M.V., Oparina N.Y., Polozov R.V., Nechipurenko Y.D., Grokhovsky S.L. (2014). Non-random DNA fragmentation in next-generation sequencing. Sci. Rep..

[127] França L.T.C., Carrilho E., Kist T.B.L. (2002). A review of DNA sequencing techniques. Q. Rev. Biophys..

[128] Ge Z., Bowles K., Goetz K., Scholl H.P.N., Wang F., Wang X., Xu S., Wang K., Wang H., Chen R. (2015). NGS-based Molecular diagnosis of 105 eyeGENE® probands with Retinitis Pigmentosa. Sci. Rep..

[129] Glöckle N., Kohl S., Mohr J., Scheurenbrand T., Sprecher A., Weisschuh N., Bernd A., Rudolph G., Schubach M., Poloschek C. (2014). Panel-based next generation sequencing as a reliable and efficient technique to detect mutations in unselected patients with retinal dystrophies. Eur. J. Hum. Genet..

[130] Farrar G.J., Carrigan M., Dockery A., Millington-Ward S., Palfi A., Chadderton N., Humphries M., Kiang A.S., Kenna P.F., Humphries P. (2017). Toward an elucidation of the molecular genetics of inherited retinal degenerations. Hum. Mol. Genet..

[131] Dockery A., Whelan L., Humphries P., Farrar G. (2021). Next-Generation Sequencing Applications for Inherited Retinal Diseases. Int. J. Mol. Sci..

[132] Rabbani B., Tekin M., Mahdieh N. (2014). The promise of whole-exome sequencing in medical genetics. J. Hum. Genet..

[133] Hart M.R., Biesecker B.B., Blout C.L., Christensen K.D., Amendola L.M., Bergstrom K.L., Biswas S., Bowling K.M., Brothers K.B., Conlin L.K. (2019). Secondary findings from clinical genomic sequencing: Prevalence, patient perspectives, family history assessment, and health-care costs from a multisite study. Genet Med..

[134] Ozsolak F. (2012). Third-generation sequencing techniques and applications to drug discovery. Expert Opin. Drug Discov..

[135] Xiao T., Zhou W. (2020). The third generation sequencing: The advanced approach to genetic diseases. Transl. Pediatr..

[136] Resta R., Biesecker B.B., Bennett R.L., Blum S., Hahn S.E., Strecker M.N., Williams J.L. (2006). A New Definition of Genetic Counseling: National Society of Genetic Counselors’ Task Force Report. J. Genet. Couns..

[137] Ciarleglio L.J., Bennett R.L., Williamson J., Mandell J.B., Marks J.H. (2003). Genetic counseling throughout the life cycle. J. Clin. Investig..

[138] Middleton A., Hall G., Patch C. (2015). Genetic counselors and Genomic Counseling in the United Kingdom. Mol. Genet. Genom. Med..

[139] Patch C., Middleton A. (2018). Genetic counselling in the era of genomic medicine. Br. Med. Bull..

[140] Strait S., Loman R., Erickson L., DeBenedictis M. (2020). Inherited retinal degeneration current genetics practices-A needs assessment. Ophthalmic Genet..

[141] McGuire A.L., Caulfield T., Cho M.K. (2008). Research ethics and the challenge of whole-genome sequencing. Nat. Rev. Genet..

[142] Shintani K., Shechtman D.L., Gurwood A.S. (2009). Review and update: Current treatment trends for patients with retinitis pigmentosa. Optom.-J. Am. Optom. Assoc..

[143] MacArthur D.G., Manolio T.A., Dimmock D.P., Rehm H.L., Shendure J., Abecasis G.R., Adams D.R., Altman R.B., Antonarakis S.E., Ashley E.A. (2014). Guidelines for investigating causality of sequence variants in human disease. Nature.

[144] Green R.C., Berg J.S., Grody W.W., Kalia S.S., Korf B.R., Martin C.L., McGuire A.L., Nussbaum R.L., O’Daniel J.M., Ormond K.E. (2013). ACMG recommendations for reporting of incidental findings in clinical exome and genome sequencing. Genet. Med..

[145] Kalia S.S., Adelman K., Bale S.J., Chung W.K., Eng C., Evans J.P., Herman G.E., Hufnagel S.B., Klein T.E., Korf B.R. (2017). Recommendations for reporting of secondary findings in clinical exome and genome sequencing, 2016 update (ACMG SF v2.0): A policy statement of the American College of Medical Genetics and Genomics. Genet. Med..

[146] Yang M., Kim J.-W. (2018). Principles of Genetic Counseling in the Era of Next-Generation Sequencing. Ann. Lab. Med..

[147] Berkman B.E., Hull S.C. (2014). The “Right Not to Know” in the Genomic Era: Time to Break From Tradition?. Am. J. Bioeth..

[148] Bennett R.L., Hampel H.L., Mandell J.B., Marks J.H. (2003). Genetic counselors: Translating genomic science into clinical practice. J. Clin. Investig..

[149] Nilsson M.P., Emmertz M., Kristoffersson U., Borg A., Larsson C., Rehn M., Winter C., Saal L.H., Brandberg Y., Loman N. (2018). Germline mutations in BRCA1 and BRCA2 incidentally revealed in a biobank research study: Experiences from re-contacting mutation carriers and relatives. J. Community Genet..

[150] Miller D.T., Lee K., Chung W.K., Gordon A.S., Herman G.E., Klein T.E., Stewart D.R., Amendola L.M., Adelman K., Bale S.J. (2021). ACMG SF v3.0 list for reporting of secondary findings in clinical exome and genome sequencing: A policy statement of the American College of Medical Genetics and Genomics (ACMG). Genet. Med..

[151] Severijns Y., de Die-Smulders C.E.M., Gültzow T., de Vries H., van Osch L.A.D.M. (2021). Hereditary diseases and child wish: Exploring motives, considerations, and the (joint) decision-making process of genetically at-risk couples. J. Community Genet..

[152] Liehr T., Lauten A., Schneider U., Schleussner E., Weise A. (2017). Noninvasive Prenatal Testing-When Is It Advantageous to Apply. Biomed. Hub.

[153] Ahmed K., Ahmed M., Potrata B., Willis T.A., Grant H.L., Allsop M.J., Hewison J., Downey L., Gale R., McKibbin M. (2015). Patient attitudes towards prenatal diagnostic testing for inherited retinal disease. Prenat. Diagn..

[154] Fesahat F., Montazeri F., Hoseini S.M. (2020). Preimplantation genetic testing in assisted reproduction technology. J. Gynecol. Obstet. Hum. Reprod..

[155] Huang X., Liu Y., Yu X., Huang Q., Lin C., Zeng J., Lan F., Wang Z. (2019). The clinical application of preimplantation genetic diagnosis for X-linked retinitis pigmentosa. J. Assist. Reprod. Genet..

[156] Greco E., Litwicka K., Minasi M.G., Cursio E., Greco P.F., Barillari P. (2020). Preimplantation Genetic Testing: Where We Are Today. Int. J. Mol. Sci..

[157] Murphy N.M., Samarasekera T.S., Macaskill L., Mullen J., Rombauts L.J.F. (2020). Genome sequencing of human in vitro fertilisation embryos for pathogenic variation screening. Sci. Rep..

[158] Hlavatá L., Ďuďáková Ľ., Trková M., Soldátová I., Skalická P., Kousal B., Lišková P. (2016). Preimplantation genetic diagnosis and monogenic inherited eye diseases. Cesk Slov Oftalmol.

[159] Cooper A.R., Jungheim E.S. (2010). Preimplantation Genetic Testing: Indications and Controversies. Clin. Lab. Med..

[160] Jackson H., Garway-Heath D., Rosen P., Bird A.C., Tuft S.J. (2001). Outcome of cataract surgery in patients with retinitis pigmentosa. Br. J. Ophthalmol..

[161] Fujiwara K., Ikeda Y., Murakami Y., Funatsu J., Nakatake S., Tachibana T., Yoshida N., Nakao S., Hisatomi T., Yoshida S. (2017). Risk Factors for Posterior Subcapsular Cataract in Retinitis Pigmentosa. Investig. Opthalmol. Vis. Sci..

[162] Yoshida N., Ikeda Y., Murakami Y., Nakatake S., Fujiwara K., Notomi S., Hisatomi T., Ishibashi T. (2015). Factors Affecting Visual Acuity after Cataract Surgery in Patients with Retinitis Pigmentosa. Ophthalmology.

[163] Liew G., Strong S., Bradley P., Severn P., Moore A.T., Webster A.R., Mitchell P., Kifley A., Michaelides M. (2019). Prevalence of cystoid macular oedema, epiretinal membrane and cataract in retinitis pigmentosa. Br. J. Ophthalmol..

[164] Yoshida N., Ikeda Y., Notomi S., Ishikawa K., Murakami Y., Hisatomi T., Enaida H., Ishibashi T. (2013). Clinical Evidence of Sustained Chronic Inflammatory Reaction in Retinitis Pigmentosa. Ophthalmology.

[165] Lu B., Yin H., Tang Q., Wang W., Luo C., Chen X., Zhang X., Lai K., Xu J., Chen X. (2020). Multiple cytokine analyses of aqueous humor from the patients with retinitis pigmentosa. Cytokine.

[166] E Chua B., Mitchell P., Cumming R. (2004). Effects of cataract type and location on visual function: The Blue Mountains Eye Study. Eye.

[167] Allen D., Vasavada A. (2006). Cataract and surgery for cataract. BMJ.

[168] Skiadaresi E., McAlinden C., Pesudovs K., Polizzi S., Khadka J., Ravalico G. (2012). Subjective Quality of Vision Before and After Cataract Surgery. Arch. Ophthalmol..

[169] van Bree M.C., Pierrache L., Zijlmans B.L., Reus N.J., Born L.I.v.D., Berg T.J.v.D. (2017). Straylight as an Indicator for Cataract Extraction in Patients with Retinal Dystrophy. Ophthalmol. Retin..

[170] van der Meulen I.J., Gjertsen J., Kruijt B., Witmer J.P., Rulo A., Schlingemann R.O., Berg T.J.V.D. (2012). Straylight measurements as an indication for cataract surgery. J. Cataract. Refract. Surg..

[171] Lundström M., Barry P., Henry Y., Rosen P., Stenevi U. (2013). Visual outcome of cataract surgery; Study from the European Registry of Quality Outcomes for Cataract and Refractive Surgery. J. Cataract. Refract. Surg..

[172] Wang J., Su F., Wang Y., Chen Y., Chen Q., Li F. (2019). Intra and post-operative complications observed with femtosecond laser-assisted cataract surgery versus conventional phacoemulsification surgery: A systematic review and meta-analysis. BMC Ophthalmol..

[173] Dikopf M.S., Chow C.C., Mieler W.F., Tu E.Y. (2013). Cataract Extraction Outcomes and the Prevalence of Zonular Insufficiency in Retinitis Pigmentosa. Am. J. Ophthalmol..

[174] Davies E.C., Pineda R. (2017). Cataract surgery outcomes and complications in retinal dystrophy patients. Can. J. Ophthalmol..

[175] Bayyoud T., Bartz-Schmidt K.U., Yoeruek E. (2013). Long-term clinical results after cataract surgery with and without capsular tension ring in patients with retinitis pigmentosa: A retrospective study. BMJ Open.

[176] Shatriah I., Jin-Poi T., Khairy-Shamel S.T., Zunaina E. (2013). Rapid anterior capsular contraction after phacoemulsification surgery in a patient with retinitis pigmentosa. Clin. Ophthalmol..

[177] Hong Y., Li H., Sun Y., Ji Y. (2020). A Review of Complicated Cataract in Retinitis Pigmentosa: Pathogenesis and Cataract Surgery. J. Ophthalmol..

[178] Nguyen X.-T., Thiadens A.A., Fiocco M., Tan W., McKibbin M., Klaver C.C., Meester-Smoor M.A., Van Cauwenbergh C., Strubbe I., Vergaro A. (2023). Outcome of Cataract Surgery in Patients With Retinitis Pigmentosa. Am. J. Ophthalmol..

[179] De Rojas J.O., Schuerch K., Mathews P.M., Cabral T., Hazan A., Sparrow J., Tsang S.H., Suh L.H. (2017). Evaluating Structural Progression of Retinitis Pigmentosa After Cataract Surgery. Am. J. Ophthalmol..

[180] Chan T.C.Y., Lam S.C., Mohamed S., Wong R.L.M. (2017). Survival analysis of visual improvement after cataract surgery in advanced retinitis pigmentosa. Eye.

[181] Nakamura Y., Mitamura Y., Hagiwara A., Kumagai K., Miura G., Sugawara T., Egawa M., Yamamoto S. (2015). Relationship between retinal microstructures and visual acuity after cataract surgery in patients with retinitis pigmentosa. Br. J. Ophthalmol..

[182] Chen C.X., Da Wang J., Zhang J.S., Xiong Y., Li J., Chen S.Y., Sun X.L., Liu Z.Y., Mayinuer Y., Wan X.H. (2021). Effect of lens capsular tension ring on preventing capsular contraction syndrome in the surgery of retinitis pigmentosa combined with cataract: Retrospective case series. Int. J. Clin. Pract..

[183] Garcia-Martin E., Rodriguez-Mena D., Dolz I., Almarcegui C., Gil-Arribas L., Bambo M.P., Larrosa J.M., Polo V., Pablo L.E. (2013). Influence of Cataract Surgery on Optical Coherence Tomography and Neurophysiology Measurements in Patients With Retinitis Pigmentosa. Am. J. Ophthalmol..

[184] Mao J., Fang D., Chen Y., Tao J., Wu M., Wu S., Wang P., Zhang Y., Shen L. (2018). Prediction of Visual Acuity After Cataract Surgery Using Optical Coherence Tomography Findings in Eyes With Retinitis Pigmentosa. Ophthalmic Surg. Lasers Imaging Retin..

[185] Chatterjee S., Agrawal D., Agrawal D., Parchand S., Sahu A. (2021). Cataract surgery in retinitis pigmentosa. Indian J. Ophthalmol..

[186] Lu Q.J., Bi J., Dd B.H., Wang D., Liu Q. (2017). Efficacy analysis of phacoemulsification combined with intraocular lens implantation in the treatment of retinitis pigmentosa complicated with cataract. Recent Adv. Ophthalmol..

[187] He H., Song H., Meng X., Cao K., Liu Y.-X., Wang J., Wan X., Jin Z.-B. (2022). Effects and Prognosis of Cataract Surgery in Patients with Retinitis Pigmentosa. Ophthalmol. Ther..

[188] Nakamura S., Fujiwara K., Yoshida N., Murakami Y., Shimokawa S., Koyanagi Y., Ikeda Y., Sonoda K.-H. (2022). Long-term Outcomes of Cataract Surgery in Patients with Retinitis Pigmentosa. Ophthalmol. Retin..

[189] Sakai D., Takagi S., Hirami Y., Nakamura M., Kurimoto Y. (2022). Use of ellipsoid zone width for predicting visual prognosis after cataract surgery in patients with retinitis pigmentosa. Eye.

[190] Leung T.W., Li R.W.-H., Kee C.-S. (2017). Blue-Light Filtering Spectacle Lenses: Optical and Clinical Performances. PLoS ONE.

[191] Masket S., Ceran B.B., Fram N.R. (2011). Spontaneous dislocation of posterior chamber intraocular lenses (PC IOLs) in patients with retinitis pigmentosa–Case series. Saudi J. Ophthalmol..

[192] Sudhir R.R., Rao S.K. (2001). Capsulorhexis phimosis in retinitis pigmentosa despite capsular tension ring implantation. J. Cataract. Refract. Surg..

[193] Najjar D.M., O Igbre A., Tsai F.F. (2012). Late capsular bag contraction and intraocular lens subluxation in retinitis pigmentosa: A case report. J. Med. Case Rep..

[194] Karahan E., Er D., Kaynak S. (2014). An Overview of Nd:YAG Laser Capsulotomy. Med. Hypothesis Discov. Innov. Ophthalmol..

[195] Strong S., Liew G., Michaelides M. (2017). Retinitis pigmentosa-associated cystoid macular oedema: Pathogenesis and avenues of intervention. Br. J. Ophthalmol..

[196] Kim Y.J., Joe S.G., Lee D.-H., Lee J.Y., Kim J.-G., Yoon Y.H. (2013). Correlations between Spectral-Domain OCT Measurements and Visual Acuity in Cystoid Macular Edema Associated with Retinitis Pigmentosa. Investig. Opthalmol. Vis. Sci..

[197] Bakthavatchalam M., Lai F.H., Rong S.S., Ng D.S., Brelen M.E. (2018). Treatment of cystoid macular edema secondary to retinitis pigmentosa: A systematic review. Surv. Ophthalmol..

[198] Hajali M., A Fishman G., Anderson R.J. (2008). The prevalence of cystoid macular oedema in retinitis pigmentosa patients determined by optical coherence tomography. Br. J. Ophthalmol..

[199] Vingolo E.M., Valente S., E Gerace E., Spadea L., Nebbioso M. (2015). Macular hole in retinitis pigmentosa patients: Microincision vitrectomy with polydimethylsiloxane as possible treatment. Eye.

[200] Hirakawa H., Iijima H., Gohdo T., Tsukahara S. (1999). Optical coherence tomography of cystoid macular edema associated with retinitis pigmentosa. Am. J. Ophthalmol..

[201] Ffytche T.J. (1972). Cystoid maculopathy in retinitis pigmentosa. Trans. Ophthalmol. Soc. United Kingd..

[202] Shahidi M., Fishman G., Ogura Y., Ambroz K., Zeimer R. (1994). Foveal thickening in retinitis pigmentosa patients with cystoid macular edema. Retina.

[203] Apushkin M.A., Fishman G.A., Janowicz M.J. (2004). Monitoring cystoid macular edema by optical coherence tomography in patients with retinitis pigmentosa. Ophthalmology.

[204] Nussenblatt R.B., Kaufman S.C., Palestine A.G., Davis M.D., Ferris F.L. (1987). Macular Thickening and Visual Acuity: Measurement in patients with cystoid macular edema. Ophthalmology.

[205] Yeo J.H., Kim Y.J., Yoon Y.H. (2020). Optical coherence tomography angiography in patients with retinitis pigmentosa–associated cystoid macular edema. Retina.

[206] Makiyama Y., Oishi A., Otani A., Ogino K., Nakagawa S., Kurimoto M., Yoshimura N. (2014). Prevalence and spatial distribution of cystoid spaces in retinitis pigmentosa: Investigation with spectral domain optical coherence tomography. Retina.

[207] Kjellström U. (2015). Reduced macular function in ABCA4 carriers. Mol. Vis..

[208] Burgess R., Millar I.D., Leroy B.P., Urquhart J.E., Fearon I.M., De Baere E., Brown P.D., Robson A.G., Wright G.A., Kestelyn P. (2008). Biallelic Mutation of BEST1 Causes a Distinct Retinopathy in Humans. Am. J. Hum. Genet..

[209] Grover S., Fishman G.A., Fiscella R.G., Adelman A.E. (1997). Efficacy of dorzolamide hydrochloride in the management of chronic cystoid macular edema in patients with retinitis pigmentosa. Retina.

[210] Fishman G.A., Gilbert L.D., Fiscella R.G., Kimura A.E., Jampol L.M. (1989). Acetazolamide for Treatment of Chronic Macular Edema in Retinitis Pigmentosa. Arch. Ophthalmol..

[211] Orzalesi N., Pierrottet C., Porta A., Aschero M. (1993). Long-term treatment of retinitis pigmentosa with acetazolamide: A pilot study. Graefe’s Arch. Clin. Exp. Ophthalmol..

[212] Liew G., Moore A.T., Webster A.R., Michaelides M. (2015). Efficacy and Prognostic Factors of Response to Carbonic Anhydrase Inhibitors in Management of Cystoid Macular Edema in Retinitis Pigmentosa. Investig. Opthalmol. Vis. Sci..

[213] Wolfensberger T.J. (1999). The role of carbonic anhydrase inhibitors in the management of macular edema. Doc. Ophthalmol..

[214] Huang Q., Chen R., Lin X., Xiang Z. (2017). Efficacy of carbonic anhydrase inhibitors in management of cystoid macular edema in retinitis pigmentosa: A meta-analysis. PLoS ONE.

[215] Grover S., Apushkin M.A., Fishman G.A. (2006). Topical Dorzolamide for the Treatment of Cystoid Macular Edema in Patients With Retinitis Pigmentosa. Am. J. Ophthalmol..

[216] Moldow B., Sander B., Larsen M., Lund-Andersen H. (1999). Effects of acetazolamide on passive and active transport of fluorescein across the normal BRB. Investig. Opthalmol. Vis. Sci..

[217] Lichter P.R. (1981). Reducing Side Effects of Carbonic Anhydrase Inhibitors. Ophthalmology.

[218] Schmickl C.N., Owens R.L., E Orr J., A Edwards B., Malhotra A. (2020). Side effects of acetazolamide: A systematic review and meta-analysis assessing overall risk and dose dependence. BMJ Open Respir. Res..

[219] Ahlstrand C., Tiselius H.-G. (1987). Urine Composition and Stone Formation During Treatment with Acetazolamide. Scand. J. Urol. Nephrol..

[220] Kass M.A., Kolker A.E., Gordon M., Goldberg I., Gieser D.K., Krupin T., Becker B. (1981). Acetazolamide and Urolithiasis. Ophthalmology.

[221] Genead M.A. (2010). Efficacy of Sustained Topical Dorzolamide Therapy for Cystic Macular Lesions in Patients With Retinitis Pigmentosa and Usher Syndrome. Arch. Ophthalmol..

[222] Ikeda Y., Yoshida N., Notomi S., Murakami Y., Hisatomi T., Enaida H., Ishibashi T. (2013). Therapeutic effect of prolonged treatment with topical dorzolamide for cystoid macular oedema in patients with retinitis pigmentosa. Br. J. Ophthalmol..

[223] Apushkin M.A., Fishman G.A., Grover S., Janowicz M.J. (2007). Rebound of cystoid macular edema with continued use of acetazolamide in patients with retinitis pigmentosa. Retina.

[224] Fishman G.A., A Apushkin M. (2007). Continued use of dorzolamide for the treatment of cystoid macular oedema in patients with retinitis pigmentosa. Br. J. Ophthalmol..

[225] Ozdemir H., Karacorlu M., Karacorlu S. (2005). Intravitreal triamcinolone acetonide for treatment of cystoid macular oedema in patients with retinitis pigmentosa. Acta Ophthalmol. Scand..

[226] Srour M., Querques G., Leveziel N., Zerbib J., Tilleul J., Boulanger-Scemama E., Souied E.H. (2013). Intravitreal dexamethasone implant (Ozurdex) for macular edema secondary to retinitis pigmentosa. Graefe’s Arch. Clin. Exp. Ophthalmol..

[227] Strong S.A., Gurbaxani A., Michaelides M. (2016). Treatment of Retinitis Pigmentosa-Associated Cystoid Macular Oedema Using Intravitreal Aflibercept (Eylea) despite Minimal Response to Ranibizumab (Lucentis): A Case Report. Case Rep. Ophthalmol..

[228] Apte R.S., Chen D.S., Ferrara N. (2019). VEGF in Signaling and Disease: Beyond Discovery and Development. Cell.

[229] Melincovici C.S., Boşca A.B., Şuşman S., Mărginean M., Mihu C., Istrate M., Moldovan I.M., Roman A.L., Mihu C.M. (2018). Vascular endothelial growth factor (VEGF)—Key factor in normal and pathological angiogenesis. Rom. J. Morphol. Embryol..

[230] Veritti D., Sarao V., De Nadai K., Chizzolini M., Parmeggiani F., Perissin L., Lanzetta P. (2020). Dexamethasone Implant Produces Better Outcomes than Oral Acetazolamide in Patients with Cystoid Macular Edema Secondary to Retinitis Pigmentosa. J. Ocul. Pharmacol. Ther..

[231] Ahn S.J., Kim K.E., Woo S.J., Park K.H. (2014). The Effect of an Intravitreal Dexamethasone Implant for Cystoid Macular Edema in Retinitis Pigmentosa: A Case Report and Literature Review. Ophthalmic Surg. Lasers Imaging Retin..

[232] Sudhalkar A., Kodjikian L., Borse N. (2017). Intravitreal dexamethasone implant for recalcitrant cystoid macular edema secondary to retinitis pigmentosa: A pilot study. Graefe’s Arch. Clin. Exp. Ophthalmol..

[233] Park U.C., Park J.H., Ma D.J., Cho I.H., Oh B.-L., Yu H.G. (2020). A randomized paired-eye trial of intravitreal dexamethasone implant for cystoid macular edema in retinitis pigmentosa. Retina.

[234] Celik N., Khoramnia R., Auffarth G.U., Sel S., Mayer C.S. (2020). Complications of dexamethasone implants: Risk factors, prevention, and clinical management. Int. J. Ophthalmol..

[235] Hagiwara A., Yamamoto S., Ogata K., Sugawara T., Hiramatsu A., Shibata M., Mitamura Y. (2011). Macular abnormalities in patients with retinitis pigmentosa: Prevalence on OCT examination and outcomes of vitreoretinal surgery. Acta Ophthalmol..

[236] Fujiwara K., Ikeda Y., Murakami Y., Nakatake S., Tachibana T., Yoshida N., Nakao S., Hisatomi T., Yoshitomi T., Sonoda K.-H. (2016). Association Between Aqueous Flare and Epiretinal Membrane in Retinitis Pigmentosa. Investig. Opthalmol. Vis. Sci..

[237] Ikeda Y., Yoshida N., Murakami Y., Nakatake S., Notomi S., Hisatomi T., Enaida H., Ishibashi T. (2015). Long-term Surgical Outcomes of Epiretinal Membrane in Patients with Retinitis Pigmentosa. Sci. Rep..

[238] Jin Z.-B., Gan D.-K., Xu G.-Z., Nao-I N. (2008). Macular Hole Formation in Patients With Retinitis Pigmentosa and Prognosis of Pars Plana Vitrectomy. Retina.

[239] Rao P.K., Shah G., Blinder K.J. (2002). Bilateral Macular Hole Formation in a Patient With Retinitis Pigmentosa. Ophthalmic Surg. Lasers Imaging Retin..

[240] García-Fernández M., Castro-Navarro J., Bajo-Fuente A. (2013). Unilateral recurrent macular hole in a patient with retinitis pigmentosa: A case report. J. Med. Case Rep..

[241] Chan W.O., Brennan N., Webster A.R., Michaelides M., Muqit M.M.K. (2020). Retinal detachment in retinitis pigmentosa. BMJ Open Ophthalmol..

[242] Dave V.P., Jalali S., Nayaka A., Pappuru R.R., Pathengay A., Das T. (2016). Clinical presentations and outcomes of rhegmatogenous retinal detachment in retinitis pigmentosa. Retina.

[243] Rishi E., Rishi P., Bhende M., Koundanya V.V., Sidramayya R., Maitray A., Rao C., Susvar P., Bhende P., Sharma T. (2018). Retinal Detachment in 31 Eyes with Retinitis Pigmentosa. Ophthalmol. Retin..

[244] Majumder P.D., Menia N., Roy R., Sen P., George A.E., Ganesh S.K., Biswas J. (2017). Uveitis in Patients with Retinitis Pigmentosa: 30 Years’ Consecutive Data. Ocul. Immunol. Inflamm..

[245] Li A.S., Pasricha M.V., Mishra K., Nguyen Q.D., Beres S.J., Wood E.H. (2022). CRB1-associated retinal dystrophy presenting as self-resolving opsoclonus and posterior uveitis. Am. J. Ophthalmol. Case Rep..

[246] Murro V., Mucciolo D.P., Sodi A., Vannozzi L., De Libero C., Simonini G., Rizzo S. (2017). Retinal capillaritis in a *CRB1*-associated retinal dystrophy. Ophthalmic Genet..

[247] Verhagen F., Kuiper J., Nierkens S., Imhof S.M., Radstake T., De Boer J. (2016). Systemic inflammatory immune signatures in a patient with CRB1 linked retinal dystrophy. Expert Rev. Clin. Immunol..

[248] Duncker T., Lee W., Jiang F., Ramachandran R., Hood D.C., Tsang S.H., Sparrow J.R., Greenstein V.C. (2018). Acute zonal occult outer retinopathy: Structural and Functional Analysis Across the Transition Zone Between Healthy and Diseased Retina. Retina.

[249] Willermain F., Greiner K., Forrester J.V. (2003). Atypical end-stage birdshot retinochoroidopathy. Ocul. Immunol. Inflamm..

[250] Chowers I., Zamir E., Banin E., Merin S. (2000). Retinitis pigmentosa associated with Fuchs’ heterochromic uveitis. Arch. Ophthalmol..

[251] Díez-Cattini G.F., Ancona-Lezama D.A., Valdés-Lara C., Morales-Cantón V. (2017). The unusual association of inverse retinitis pigmentosa and Fuchs’ heterochromic iridocyclitis. Int. J. Retin. Vitr..

[252] Born L.I.V.D., van Schooneveld M.J., de Jong P.T., Bleeker-Wagemakers E.M. (1994). Fuchs’ heterochromic uveitis associated with retinitis pigmentosa in a father and son. Br. J. Ophthalmol..

[253] Vuorre I., Saari M., Tiilikainen A., Rasanen O. (1979). Fuchs’ heterochromic cyclitis associated with retinitis pigmentosa: A family study. Can. J. Ophthalmol..

[254] Yalvaç I.S., Altintas A.K., Gökdere A., Duman S. (1998). Fuchs’ heterochromic uveitis associated with retinitis pigmentosa. Acta Ophthalmol. Scand..

[255] Sandinha T. (2003). Retinitis pigmentosa associated with Fuchs’ heterochromic uveitis. Eye.

[256] Lichtinger A., Chowers I., Amer R. (2010). Usher syndrome associated with Fuchs’ heterochromic uveitis. Graefe’s Arch. Clin. Exp. Ophthalmol..

[257] Sevgi D.D., Davoudi S., Comander J., Sobrin L. (2017). Retinal pigmentary changes in chronic uveitis mimicking retinitis pigmentosa. Graefe’s Arch. Clin. Exp. Ophthalmol..

[258] Hettinga Y.M., van Genderen M.M., Wieringa W., Norel J.O.-V., de Boer J.H. (2016). Retinal Dystrophy in 6 Young Patients Who Presented with Intermediate Uveitis. Ophthalmology.

[259] Szabó E., Brichová M., Lišková P., Svozílková P., Ríhová E. (2013). Retinitis pigmentosa mimicking uveitis. A case report. Cesk Slov. Oftalmol..

[260] Latorre R.H., Fernandez-perez S., Garcia-martin E., Satue M., Idoipe M., De La Mata G., Torrón C. (2012). Bilateral intermediate uveitis asociated with retinosis pigmentosa. Acta Ophthalmol..

[261] Kaufman M., Medina-Mendez C., Friberg T., Eller A. (2013). Evaluation of peripheral retinal vasculitis in retinitis pigmentosa using wide-field fluorescein angiography. Investig. Ophthalmol. Vis. Sci..

[262] Badeeb O., Trope G., Musarella M. (1993). Primary angle closure glaucoma and retinitis pigmentosa. Acta Ophthalmol..

[263] Hung M.-C., Chen Y.-Y. (2022). Association between retinitis pigmentosa and an increased risk of primary angle closure glaucoma: A population-based cohort study. PLoS ONE.

[264] Pradhan C., Khadka S., Joshi P. (2020). Angle Closure Glaucoma in Retinitis Pigmentosa. Case Rep. Ophthalmol. Med..

[265] Lai J., Choy B.N.K., Shum J.W.H. (2016). Management of Primary Angle-Closure Glaucoma. Asia-Pacific J. Ophthalmol..

[266] Slade A., Isa F., Kyte D., Pankhurst T., Kerecuk L., Ferguson J., Lipkin G., Calvert M. (2018). Patient reported outcome measures in rare diseases: A narrative review. Orphanet J. Rare Dis..

[267] Wilkinson M.E., Shahid K.S. (2018). Low vision rehabilitation: An update. Saudi J. Ophthalmol..

[268] Langelaan M., de Boer M.R., van Nispen R.M., Wouters B., Moll A.C., van Rens G.H. (2009). Change in quality of life after rehabilitation: Prognostic factors for visually impaired adults. Int. J. Rehabil. Res..

[269] van Nispen R.M., Virgili G., Hoeben M., Langelaan M., Klevering J., Keunen J.E., van Rens G.H. (2020). Low vision rehabilitation for better quality of life in visually impaired adults. Cochrane Database Syst. Rev..

[270] Owsley C. (2009). Characteristics of Low-Vision Rehabilitation Services in the United States. Arch. Ophthalmol..

[271] Lamoureux E.L., Pallant J.F., Pesudovs K., Rees G., Hassell J.B., Keeffe J.E. (2007). The Effectiveness of Low-Vision Rehabilitation on Participation in Daily Living and Quality of Life. Investig. Opthalmol. Vis. Sci..

[272] Stelmack J.A., Tang X.C., Wei Y., Massof R.W., for the Low-Vision Intervention Trial Study Group (2012). The Effectiveness of Low-Vision Rehabilitation in 2 Cohorts Derived From the Veterans Affairs Low-Vision Intervention Trial. JAMA Ophthalmol..

[273] Massof R.W., Ahmadian L., Grover L.L., Deremeik J.T., Goldstein J.E., Rainey C., Epstein C., Barnett G.D. (2007). The Activity Inventory: An Adaptive Visual Function Questionnaire. Optom. Vis. Sci..

[274] Mangione C.M., Lee P., Gutierrez P.R., Spritzer K., Berry S., Hays R.D. (2001). Development of the 25-list-item National Eye Institute Visual Function Questionnaire. Arch. Ophthalmol..

[275] Bruijning J., Van Nispen R., Verstraten P., Van Rens G. (2010). A Dutch ICF Version of the Activity Inventory: Results from Focus Groups with Visually Impaired Persons and Experts. Ophthalmic Epidemiol..

[276] Lacy G.D., Abalem M.F., Andrews C.A., Popova L.T., Santos E.P., Yu G., Rakine H.Y., Baig N., Ehrlich J.R., Fahim A.T. (2020). The Michigan Retinal Degeneration Questionnaire: A Patient-Reported Outcome Instrument for Inherited Retinal Degenerations. Am. J. Ophthalmol..

[277] Virgili G., Acosta R., Bentley S.A., Giacomelli G., Allcock C., Evans J.R. (2018). Reading aids for adults with low vision. Cochrane Database Syst. Rev..

[278] Nguyen X., Koopman J., Genderen M.M., Stam H.L., Boon C.J. (2021). Artificial vision: The effectiveness of the OrCam in patients with advanced inherited retinal dystrophies. Acta Ophthalmol..

[279] Lorenzini M.-C., Wittich W. (2020). Factors related to the use of magnifying low vision aids: A scoping review. Disabil. Rehabil..

[280] Wittich W., Lorenzini M.-C., Markowitz S.N., Tolentino M., Gartner S.A., Goldstein J.E., Dagnelie G. (2018). The Effect of a Head-mounted Low Vision Device on Visual Function. Optom. Vis. Sci..

[281] Markowitz M. (2006). Occupational therapy interventions in low vision rehabilitation. Can. J. Ophthalmol..

[282] Scott A.W., Bressler N.M., Ffolkes S., Wittenborn J.S., Jorkasky J. (2016). Public Attitudes About Eye and Vision Health. JAMA Ophthalmol..

[283] Demmin D.L., Silverstein S.M. (2020). Visual Impairment and Mental Health: Unmet Needs and Treatment Options. Clin. Ophthalmol..

[284] Munaw M.B., Tegegn M.T. (2022). Visual impairment and psychological distress among adults attending the University of Gondar tertiary eye care and training center, Northwest Ethiopia: A comparative cross-sectional study. PLoS ONE.

[285] Horowitz A., Leonard R., Reinhardt J.P. (2000). Measuring Psychosocial and Functional Outcomes of a Group Model of Vision Rehabilitation Services for Older Adults. J. Vis. Impair. Blind..

[286] Rees G., Ponczek E., Hassell J., E Keeffe J., Lamoureux E.L. (2010). Psychological outcomes following interventions for people with low vision: A systematic review. Expert Rev. Ophthalmol..

[287] Veldman M.H.J., van der Aa H.P.A., Bode C., Knoop H., Hulshof C.T.J., Koopmanschap M., Stavleu E., van Rens G.H.M.B., van Nispen R.M.A. (2021). E-nergEYEze, a vision-specific eHealth intervention based on cognitive behavioral therapy and self-management to reduce fatigue in adults with visual impairment: Study protocol for a randomized controlled trial. Trials.

[288] Virgili G., Rubin G. (2010). Orientation and mobility training for adults with low vision. Cochrane Database Syst. Rev..

[289] Zijlstra G.R., Ballemans J., Kempen G.I. (2013). Orientation and mobility training for adults with low vision: A new standardized approach. Clin. Rehabil..

[290] Li C., Samulski R.J. (2020). Engineering adeno-associated virus vectors for gene therapy. Nat. Rev. Genet..

[291] Bulcha J.T., Wang Y., Ma H., Tai P.W.L., Gao G. (2021). Viral vector platforms within the gene therapy landscape. Signal Transduct. Target. Ther..

[292] Cideciyan A.V., Sudharsan R., Dufour V.L., Massengill M.T., Iwabe S., Swider M., Lisi B., Sumaroka A., Marinho L.F., Appelbaum T. (2018). Mutation-independent rhodopsin gene therapy by knockdown and replacement with a single AAV vector. Proc. Natl. Acad. Sci. USA.

[293] DiCarlo J.E., Mahajan V.B., Tsang S.H. (2018). Gene therapy and genome surgery in the retina. J. Clin. Investig..

[294] Botto C., Rucli M., Tekinsoy M.D., Pulman J., Sahel J.-A., Dalkara D. (2022). Early and late stage gene therapy interventions for inherited retinal degenerations. Prog. Retin. Eye Res..

[295] Leroy B.P., Fischer M.D., Flannery J.G., MacLaren R.E., Dalkara D., Scholl H.P., Chung D.C., Spera C., Viriato D., Banhazi J. (2022). Gene therapy for inherited retinal disease: Long-term durability of effect. Ophthalmic Res..

[296] Gange W.S., Sisk R.A., Besirli C.G., Lee T.C., Havunjian M., Schwartz H., Borchert M., Sengillo J.D., Mendoza C., Berrocal A.M. (2022). Perifoveal Chorioretinal Atrophy after Subretinal Voretigene Neparvovec-rzyl for RPE65-Mediated Leber Congenital Amaurosis. Ophthalmol. Retin..

[297] Reichel F.F., Seitz I., Wozar F., Dimopoulos S., Jung R., Kempf M., Kohl S., Kortüm F.C., Ott S., Pohl L. (2022). Development of retinal atrophy after subretinal gene therapy with voretigene neparvovec. Br. J. Ophthalmol..

[298] Peng Y., Tang L., Zhou Y. (2017). Subretinal Injection: A Review on the Novel Route of Therapeutic Delivery for Vitreoretinal Diseases. Ophthalmic Res..

[299] Ghoraba H.H., Akhavanrezayat A., Karaca I., Yavari N., Lajevardi S., Hwang J., Regenold J., Matsumiya W., Pham B., Zaidi M. (2022). Ocular Gene Therapy: A Literature Review with Special Focus on Immune and Inflammatory Responses. Clin. Ophthalmol..

[300] Boon N., Wijnholds J., Pellissier L.P. (2020). Research Models and Gene Augmentation Therapy for CRB1 Retinal Dystrophies. Front. Neurosci..

[301] Bansal M., Acharya S., Sharma S., Phutela R., Rauthan R., Maiti S., Chakraborty D. (2021). CRISPR Cas9 based genome editing in inherited retinal dystrophies. Ophthalmic Genet..

[302] Burnight E.R., Giacalone J.C., Cooke J.A., Thompson J.R., Bohrer L.R., Chirco K.R., Drack A.V., Fingert J.H., Worthington K.S., Wiley L.A. (2018). CRISPR-Cas9 genome engineering: Treating inherited retinal degeneration. Prog. Retin. Eye Res..

[303] Pulman J., Sahel J.-A., Dalkara D. (2022). New Editing Tools for Gene Therapy in Inherited Retinal Dystrophies. CRISPR J..

[304] Rasul M.F., Hussen B.M., Salihi A., Ismael B.S., Jalal P.J., Zanichelli A., Jamali E., Baniahmad A., Ghafouri-Fard S., Basiri A. (2022). Strategies to overcome the main challenges of the use of CRISPR/Cas9 as a replacement for cancer therapy. Mol. Cancer.

[305] Collin R.W., Garanto A. (2017). Applications of antisense oligonucleotides for the treatment of inherited retinal diseases. Curr. Opin. Ophthalmol..

[306] Girach A., Audo I., Birch D.G., Huckfeldt R.M., Lam B.L., Leroy B.P., Michaelides M., Russell S.R., Sallum J.M., Stingl K. (2022). RNA-based therapies in inherited retinal diseases. Ther. Adv. Ophthalmol..

[307] Collin R.W., Den Hollander A.I., van der Velde-Visser S.D., Bennicelli J., Bennett J., Cremers F.P. (2012). Antisense Oligonucleotide (AON)-based Therapy for Leber Congenital Amaurosis Caused by a Frequent Mutation in CEP290. Mol. Ther.-Nucleic Acids.

[308] Duebel J., Marazova K., Sahel J.-A. (2015). Optogenetics. Curr. Opin. Ophthalmol..

[309] Sakai D., Tomita H., Maeda A. (2022). Optogenetic Therapy for Visual Restoration. Int. J. Mol. Sci..

[310] Sahel J.-A., Boulanger-Scemama E., Pagot C., Arleo A., Galluppi F., Martel J.N., Degli Esposti S., Delaux A., de Saint Aubert J.-B., de Montleau C. (2021). Partial recovery of visual function in a blind patient after optogenetic therapy. Nat. Med..

[311] Shen Y. (2020). Stem cell therapies for retinal diseases: From bench to bedside. J. Mol. Med..

[312] Bacakova L., Zarubova J., Travnickova M., Musilkova J., Pajorova J., Slepicka P., Kasalkova N.S., Svorcik V., Kolska Z., Motarjemi H. (2018). Stem cells: Their source, potency and use in regenerative therapies with focus on adipose-derived stem cells–a review. Biotechnol. Adv..

[313] Sharma A., Jaganathan B.G. (2021). Stem Cell Therapy for Retinal Degeneration: The Evidence to Date. Biol. Targets Ther..

[314] Tuekprakhon A., Sangkitporn S., Trinavarat A., Pawestri A.R., Vamvanij V., Ruangchainikom M., Luksanapruksa P., Pongpaksupasin P., Khorchai A., Dambua A. (2021). Intravitreal autologous mesenchymal stem cell transplantation: A non-randomized phase I clinical trial in patients with retinitis pigmentosa. Stem Cell Res. Ther..

[315] Gagliardi G., Ben M’Barek K., Goureau O. (2019). Photoreceptor cell replacement in macular degeneration and retinitis pigmentosa: A pluripotent stem cell-based approach. Prog. Retin. Eye Res..

[316] Siqueira R.C. (2012). Stem cell therapy in retinal diseases?. Rev. Bras. Hematol. Hemoter..

[317] Tang Z., Zhang Y., Wang Y., Zhang D., Shen B., Luo M., Gu P. (2017). Progress of stem/progenitor cell-based therapy for retinal degeneration. J. Transl. Med..

[318] Blum B., Benvenisty N. (2008). The Tumorigenicity of Human Embryonic Stem Cells. Adv. Cancer Res..

[319] Brown C., Agosta P., McKee C., Walker K., Mazzella M., Alamri A., Svinarich D., Chaudhry G.R. (2022). Human primitive mesenchymal stem cell-derived retinal progenitor cells improved neuroprotection, neurogenesis, and vision in rd12 mouse model of retinitis pigmentosa. Stem Cell Res. Ther..

[320] Liu Y., Chen S.J., Li S.Y., Qu L.H., Meng X.H., Wang Y., Xu H.W., Liang Z.Q., Yin Z.Q. (2017). Long-term safety of human retinal progenitor cell transplantation in retinitis pigmentosa patients. Stem Cell Res. Ther..

[321] Singh M.S., Park S.S., Albini T.A., Canto-Soler M.V., Klassen H., MacLaren R.E., Takahashi M., Nagiel A., Schwartz S.D., Bharti K. (2020). Retinal stem cell transplantation: Balancing safety and potential. Prog. Retin. Eye Res..

[322] Musiał-Wysocka A., Kot M., Majka M. (2019). The Pros and Cons of Mesenchymal Stem Cell-Based Therapies. Cell Transplant..

[323] Ayton L.N., Barnes N., Dagnelie G., Fujikado T., Goetz G., Hornig R., Jones B.W., Muqit M.M., Rathbun D.L., Stingl K. (2020). An update on retinal prostheses. Clin. Neurophysiol..

[324] Miyoshi T., Morimoto T., Sawai H., Fujikado T. (2021). Spatial Resolution of Suprachoroidal–Transretinal Stimulation Estimated by Recording Single-Unit Activity From the Cat Lateral Geniculate Nucleus. Front. Neurosci..

[325] Shim S., Eom K., Jeong J., Kim S.J. (2020). Retinal Prosthetic Approaches to Enhance Visual Perception for Blind Patients. Micromachines.

[326] Ayton L.N., Blamey P.J., Guymer R.H., Luu C.D., Nayagam D.A.X., Sinclair N.C., Shivdasani M.N., Yeoh J., McCombe M.F., Briggs R.J. (2014). First-in-Human Trial of a Novel Suprachoroidal Retinal Prosthesis. PLoS ONE.

[327] Fujikado T., Kamei M., Sakaguchi H., Kanda H., Morimoto T., Ikuno Y., Nishida K., Kishima H., Maruo T., Konoma K. (2011). Testing of Semichronically Implanted Retinal Prosthesis by Suprachoroidal-Transretinal Stimulation in Patients with Retinitis Pigmentosa. Investig. Opthalmol. Vis. Sci..

[328] White J., Knight L., da Cruz L., E Stanga P., Patrick H., Powell H., Berry L., Withers K., Carolan-Rees G., Jackson T.L. (2021). Effects of the Argus II Retinal Prosthesis System on the Quality of Life of Patients With Ultra-Low Vision Due to Retinitis Pigmentosa: Protocol for a Single-Arm, Mixed Methods Study. JMIR Res. Protoc..

[329] Chuang A.T., E Margo C., Greenberg P.B. (2014). Retinal implants: A systematic review. Br. J. Ophthalmol..

[330] Stronks H.C., Dagnelie G. (2014). The functional performance of the Argus II retinal prosthesis. Expert Rev. Med. Devices.

[331] Sieving P.A., Caruso R.C., Tao W., Coleman H.R., Thompson D.J.S., Fullmer K.R., Bush R.A. (2006). Ciliary neurotrophic factor (CNTF) for human retinal degeneration: Phase I trial of CNTF delivered by encapsulated cell intraocular implants. Proc. Natl. Acad. Sci. USA.

[332] Birch D.G., Weleber R.G., Duncan J.L., Jaffe G.J., Tao W. (2013). Randomized Trial of Ciliary Neurotrophic Factor Delivered by Encapsulated Cell Intraocular Implants for Retinitis Pigmentosa. Am. J. Ophthalmol..

[333] Birch D.G., Bennett L.D., Duncan J.L., Weleber R.G., Pennesi M.E. (2016). Long-term Follow-up of Patients With Retinitis Pigmentosa Receiving Intraocular Ciliary Neurotrophic Factor Implants. Am. J. Ophthalmol..

[334] Campochiaro P.A., Iftikhar M., Hafiz G., Akhlaq A., Tsai G., Wehling D., Lu L., Wall G.M., Singh M.S., Kong X. (2020). Oral N-acetylcysteine improves cone function in retinitis pigmentosa patients in phase I trial. J. Clin. Investig..

[335] Kong X., Hafiz G., Wehling D., Akhlaq A., Campochiaro P.A. (2021). Locus-Level Changes in Macular Sensitivity in Patients with Retinitis Pigmentosa Treated with Oral N-acetylcysteine. Am. J. Ophthalmol..

[336] Maccarone R., Tisi A., Passacantando M., Ciancaglini M. (2020). Ophthalmic Applications of Cerium Oxide Nanoparticles. J. Ocul. Pharmacol. Ther..

[337] Wong L.L., Pye Q.N., Chen L., Seal S., McGinnis J.F. (2015). Defining the Catalytic Activity of Nanoceria in the P23H-1 Rat, a Photoreceptor Degeneration Model. PLoS ONE.

[338] Berson E.L., Rosner B., Sandberg M.A., Hayes K.C., Nicholson B.W., Weigel-DiFranco C., Willett W. (1993). A Randomized Trial of Vitamin A and Vitamin E Supplementation for Retinitis Pigmentosa. Arch. Ophthalmol..

[339] Berson E.L., Rosner B., Sandberg M.A., Weigel-DiFranco C., Moser A., Brockhurst R.J., Hayes K.C., Johnson C., Anderson E.J., Gaudio A.R. (2004). Further Evaluation of Docosahexaenoic Acid in Patients With RetinitisPigmentosa Receiving Vitamin A Treatment: Subgroup analyses. Arch. Ophthalmol..

[340] Berson E.L., Weigel-DiFranco C., Rosner B., Gaudio A.R., Sandberg M.A. (2018). Association of Vitamin A Supplementation With Disease Course in Children With Retinitis Pigmentosa. JAMA Ophthalmol..

[341] Klaver C.C.W., Thiadens A.A.H.J. (2018). Vitamin A for Children With Retinitis Pigmentosa: An Unresolved Mystery. JAMA Ophthalmol..

[342] Sajovic J., Meglič A., Glavač D., Markelj Š., Hawlina M., Fakin A. (2022). The Role of Vitamin A in Retinal Diseases. Int. J. Mol. Sci..

[343] Hoffman D.R., Hughbanks-Wheaton D.K., Pearson N.S., Fish G.E., Spencer R., Takacs A., Klein M., Locke K.G., Birch D. (2014). Four-Year Placebo-Controlled Trial of Docosahexaenoic Acid in X-Linked Retinitis Pigmentosa (DHAX Trial): A randomized clinical trial. JAMA Ophthalmol..

[344] Hoffman D.R., Hughbanks-Wheaton D.K., Spencer R., Fish G.E., Pearson N.S., Wang Y.-Z., Klein M., Takacs A., Locke K.G., Birch D.G. (2015). Docosahexaenoic Acid Slows Visual Field Progression in X-Linked Retinitis Pigmentosa: Ancillary Outcomes of the DHAX Trial. Investig. Opthalmol. Vis. Sci..

[345] Rayapudi S., Schwartz S.G., Wang X., Chavis P. (2013). Vitamin A and fish oils for retinitis pigmentosa. Cochrane Database Syst. Rev..

[346] Schwartz S.G., Wang X., Chavis P., E Kuriyan A., A Abariga S. (2020). Vitamin A and fish oils for preventing the progression of retinitis pigmentosa. Cochrane Database Syst. Rev..

[347] Zhao Y., Feng K., Liu R., Pan J., Zhang L., Lu X. (2019). Vitamins and Mineral Supplements for Retinitis Pigmentosa. J. Ophthalmol..

[348] Massof R.W., A Fishman G. (2010). How Strong Is the Evidence That Nutritional Supplements Slow the Progression of Retinitis Pigmentosa?. Arch. Ophthalmol..

[349] Sofi F., Sodi A., Franco F., Murro V., Biagini D., Miele A., Abbruzzese G., Mucciolo D.P., Virgili G., Menchini U. (2016). Dietary profile of patients with Stargardt’s disease and Retinitis Pigmentosa: Is there a role for a nutritional approach?. BMC Ophthalmol..

[350] Radu R.A., Yuan Q., Hu J., Peng J.H., Lloyd M., Nusinowitz S., Bok D., Travis G.H. (2008). Accelerated Accumulation of Lipofuscin Pigments in the RPE of a Mouse Model for *ABCA4*-Mediated Retinal Dystrophies following Vitamin A Supplementation. Investig. Opthalmol. Vis. Sci..

[351] Federspiel C.A., Bertelsen M., Kessel L. (2018). Vitamin A in Stargardt disease—An evidence-based update. Ophthalmic Genet..

