# Supplementary material for: Retinitis Pigmentosa: Current Clinical Management and Emerging Therapies
Source: Int J Mol Sci. 2023 Apr 19;24(8):7481. doi: 10.3390/ijms24087481 (PMC10139437; doi:10.3390/ijms24087481)
Supplement: Supplementary file 1 [file ijms-24-07481-s001.zip › ijms-2307663-supplementary.pdf]

Supplemental Table S1. Overview of studies involving patients with retinitis pigmentosa that underwent surgery for retinal detachments

	Chan et al. 2020	Dave et al. 2016	Rishi et al. 2018
**Sample size**	90 patients	17 patients	31 patients
**Follow-up**	15.4 years	8.52 ± 12.6	33 months (range 1-145)
**Mean Age**	Mean age 32.8 years	Mean age: 34.5 ± 16.4	Mean age 22 years
**Type of retinal detachment**	Rhegmatogenous (68%)<br>Exudative (21%)<br>Tractional (3%)<br>Combined (8%)	Rhegmatogenous (100%)	N/A
**Intervention**	Scleral buckling (3%)<br>PPV (20%)<br>Unknown surgical intervention (39%)*<br>Conservative (28%)<br>Not treated (10%)	Scleral buckling (30%)<br>PPV (35%)<br>Not treated (35%)	Scleral buckling (42%)<br>PPV (32%)<br>Not treated (26%)
**Final attachment rate**	85%	91%	96%
**Change in BCVA**	1.52 vs 1.25 logMAR ($p = 0.098$)	1.4 ± 0.9 vs 1.1 ± 0.8 logMAR ($p = 0.15$)	1.63 ± 0.89 vs 0.87 ± 0.25 logMAR ($p<0.001$)
**Complications**	Re-RD (n = 5)<br>High IOP (n = 3)<br>UGH (n = 1)<br>Scleritis (n = 1)<br>IOL subluxation (n = 1)	N/A	Re-RD (n = 3)<br>Epiretinal membrane (n = 3);<br>cataract (n = 2);<br>Macular hole (n = 1);<br>High IOP (n = 1)

BCVA = best-corrected visual acuity; PPV = pars plana vitrectomy; IOP = intraocular pressure; UGH = uveitis-glaucoma-hyphaema syndrome; IOL = intraocular lens; re-RD = retinal re-detachment

*No information was available for the remaining surgical interventions in this study by Chan et al. Patients with exudative retinal detachments were treated conservatively. Patients that were not treated were due to poor visual prognosis.
